# Partially Reduced and Stabilized Phase‐Changed MoS_2_ Hybrids for Vapor Molecular Absorption at Defects in Edge Sites

**DOI:** 10.1002/smsc.202500605

**Published:** 2026-04-29

**Authors:** Hye Gyu Cha, Taeseo Ko, Taehyeon Kim, Yun Ji Hwang, Sushanta Kumar Das, Kyoungmin Min, Seong Chan Jun

**Affiliations:** ^1^ School of Mechanical Engineering Yonsei University Seoul Republic of Korea

**Keywords:** field effect transistor sensor, gas sensing, molybdenum disulfide, partial reduction, phase change

## Abstract

Current research in gas sensor technology emphasizes developing high‐performance, miniaturized devices that operate at room temperature. Among emerging materials, molybdenum disulfide (MoS_2_), a layered semiconductor, has garnered significant attention for its ability to detect diverse analytes with a high surface to volume ratio. In this study, composites of 1T and 2H MoS_2_ with reduced graphene oxide (rGO) were synthesized, combining distinct physical and chemical properties that enable unique interactions with gas molecules. The applied synthesis routes are cost effective, reproducible, and readily compatible with field effect transistor printed devices. Sensor performance for nitric oxide (NO), nitrogen dioxide (NO_2_), and ammonia (NH_3_) gases in 1T‐based hybrids was superior to that in 2H‐based hybrids, which was attributed to the metallic and hydrophilic nature of the 1T phase. The hybrids displayed excellent performance across a wide concentration ranging from 500 ppb to 2 ppm. Notably, for NO detection, the 1T MoS_2_@rGO sensor achieved responses of 5.4% at 500 ppb and 38.1% at 2 ppm. Density functional theory further confirmed the metallic character of the 1T phase. These results underscore the promise of phase‐engineered MoS_2_@rGO hybrids as next‐generation materials for reliable room temperature gas sensing technologies.

## Graphical abstract

Schematic illustration of field effect transistor‐type sensor electrode fabrication and synthesis of MoS_2_@rGO hybrids. 1T or 2H MoS_2_ were combined with GO, followed by thermal partial reduction to form MoS_2_@rGO with defect sites. The removal of hydroxyl groups as moisture increased specific surface area and porous size, enhancing vapor adsorption. Comparative sensing performance of 1T MoS_2_@rGO, rGO, and 2H MoS_2_@rGO is demonstrated.

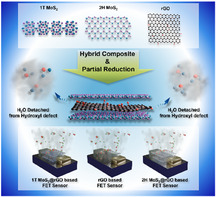

## 1 Introduction

Accurate detection of hazardous gases such as NH_3_, NO, and NO_2_ is critical in various fields, including environmental monitoring, industrial safety, and public health [1]. These gases are commonly emitted during combustion, agricultural activities, vehicle exhaust, and chemical manufacturing. Even at low concentrations, they can pose significant risks to air quality and human well‐being [2, 3]. In many industrial and urban environments, low‐level nitrogen‐based gases pose critical safety and process‐control challenges because they are often imperceptible to humans yet capable of causing cumulative health risks or equipment degradation. In particular, semiconductor and display cleanrooms, chemical process facilities, and agricultural storage environments require continuous monitoring of NO_*x*_ and NH_3_ at sub‐ppm to low‐ppm levels to prevent long‐term exposure and ensure operational stability, as summarized in Table S1. Based on these application‐driven requirements, a detection range of 0.5–2 ppm was deliberately selected in this study.

Among them, NH_3_, NO, and NO_2_ are of particular interest because of their high reactivity, toxicity, and their role as key indicators in air pollution monitoring systems [4, 5, 6]. Ammonia is a prevalent alkaline gas that originates from both natural and anthropogenic sources and contributes to secondary particulate matter formation [4]. Nitric oxide and nitrogen dioxide, collectively referred to as NO_*x*_ gases, are major byproducts of fossil fuel combustion and are regulated pollutants due to their role in ozone formation and respiratory irritation [5, 6].

However, the selective detection of NO and NO_2_, especially when present simultaneously in complex gas mixtures, remains technically challenging [7]. Both are oxidizing gases with similar physicochemical properties, such as comparable molecular sizes, diffusion rates, and electron‐withdrawing tendencies [8, 9]. Furthermore, NO can be rapidly oxidized to NO_2_ in ambient conditions, resulting in dynamic interconversion between the two species [10, 11]. This redox coupling leads to signal interference and complicates the discrimination of individual gas contributions in sensor responses [12]. Hence, achieving high selectivity for either NO or NO_2_ in mixed gas environments requires finely tuned sensor materials with distinct adsorption energetics and charge transfer mechanisms for each gas.

To detect these hazardous gases with high precision at trace levels, nanomaterial‐based gas sensors, especially those utilizing 2D layered materials, are attracting increasing attention [13, 14]. Among them, reduced graphene oxide (rGO) and molybdenum disulfide (MoS_2_) are regarded as promising active materials because of their outstanding physicochemical properties and high surface reactivity [13, 14, 15].

The reduced form of graphene oxide, with a large specific surface area, abundant oxygen‐containing functional groups (e.g., –OH, –COOH), and enhanced electrical conductivity, contributes to strong interactions with gas molecules [16, 17, 18, 19]. However, rGO suffers from limitations such as low selectivity and reduced long‐term stability under varying environmental conditions [20]. However, MoS_2_, a layered transition metal dichalcogenide, can exist in two polymorphs with distinct properties: a metallic 1T phase and semiconducting 2H phase [21]. 1T MoS_2_ exhibits high electrical conductivity and a high density of active sites, making it highly responsive to polar gases such as NH_3_, NO_2,_ and NO [22, 23]. It also offers fast response and recovery times. Nonetheless, the 1T phase is thermodynamically unstable and tends to revert to the more stable 2H phase over time [24, 25, 26]. In contrast, 2H MoS_2_ offers better chemical stability, higher gas selectivity, and long‐term sensor reliability, particularly for gases like NO_2,_ [27, 28] but suffers from slower response times and lower electrical conductivity [29].

To overcome these trade‐offs, hybrid structures combining MoS_2_ and rGO have been actively explored [30, 31, 32, 33, 34]. These MoS_2_@rGO heterostructures combine the high charge mobility of rGO with the selective gas adsorption of MoS_2_, resulting in synergistic enhancements in sensitivity, selectivity, response speed, and device stability [30, 31, 32]. For instance, MoS_2_@rGO hybrids have demonstrated improved NH_3_ sensing at room temperature [33], along with fast recovery and high‐performance NO and NO_2_ detection at low thresholds [34]. Furthermore, by tailoring the phase MoS_2_ (1T vs. 2H) and composition ratio, the sensing behavior can be optimized for specific target gases particularly those involved in carcinogenic pathways.

In this work, we demonstrate MoS_2_@rGO hybrid structures integrated into field‐effect transistor (FET) devices for the detection of nitrogen‐based gases (NH_3_, NO, and NO_2_), highlighting their phase‐dependent sensing performance.

In summary, rGO, 1T MoS_2_, 2H MoS_2_, and their hybrids represent a highly promising platform for the development of next‐generation, high‐performance gas sensors in detecting environmental carcinogens and contributing to cancer prevention through early monitoring.

## 2 Results and Discussion

### 2.1 Nitrogen‐Based Vapor Sensing Process of MoS_2_ Hybrid Composite Active Material

**FIGURE 1:**
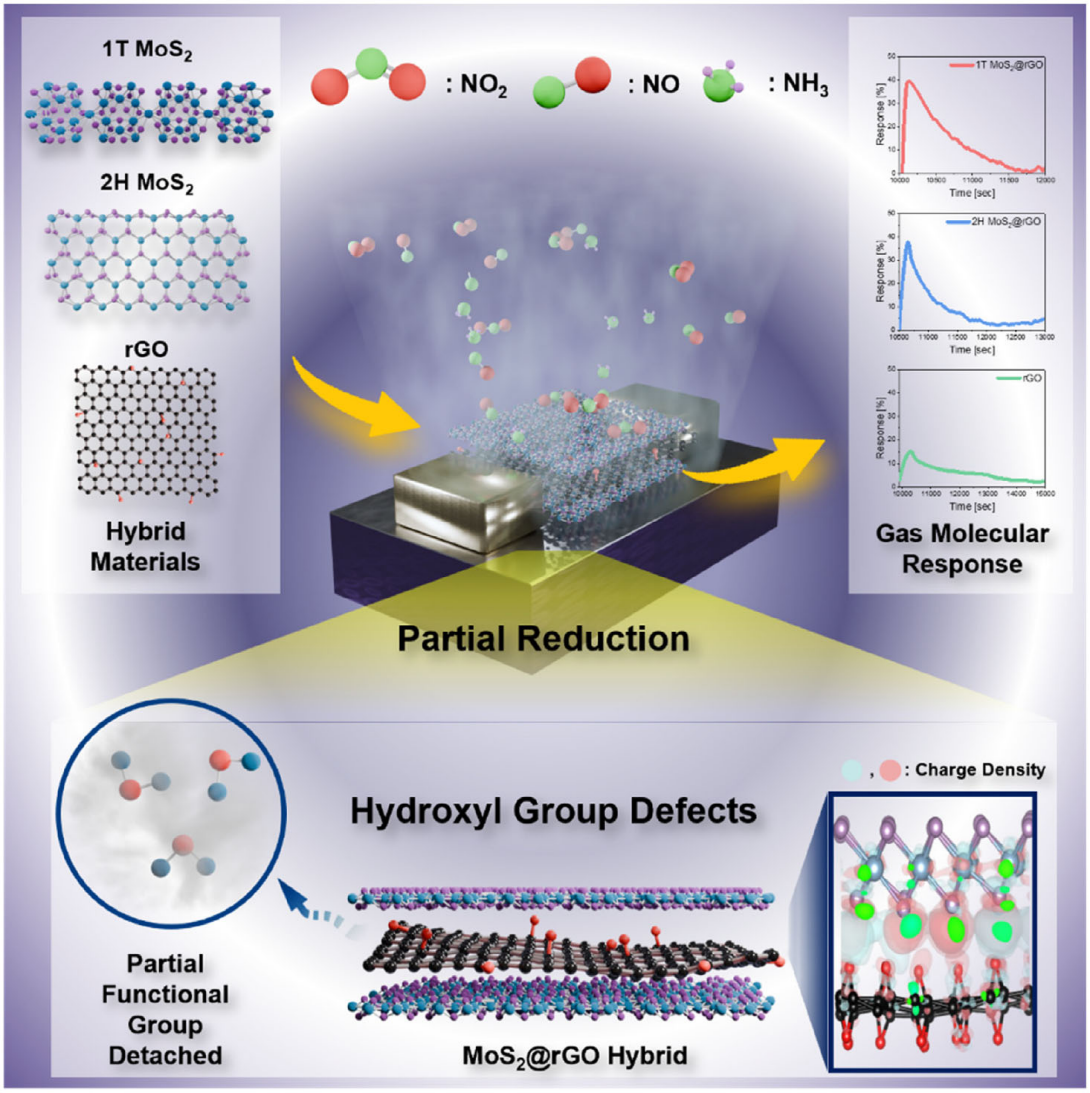
Schematic of gas adsorption on sensing layers (1T MoS_2_@rGO, 2H MoS_2_@rGO, and rGO). Hybrid sensing layers of 1T MoS_2_, 2H MoS_2_, and rGO with partial hydroxyl group defects exhibit NO, NO_2_, and NH_3_ adsorption and corresponding FET sensor responses.

Figure 1 presents a schematic representation of the MoS_2_@rGO hybrid gas sensing concept. In this platform, reduced graphene oxide (rGO) is hybridized with both 1T and 2H MoS_2_, and the partial reduction introduces hydroxyl group defects that serve as active adsorption sites. In addition, unsaturated edge sites generated during partial reduction play a crucial role in facilitating gas adsorption and charge transfer. The incorporation of the 1T phase is particularly critical, as its metallic conductivity and defect‐rich nature enable efficient charge transfer and strengthen electronic coupling at the MoS_2_@rGO interface [35, 36, 37, 38]. Each interdigitated electrode (IDE) sensing region has a lateral dimension of approximately 0.95 mm × 0.95 mm, while the source and drain contact pads have a lateral dimension of approximately 0.6 mm × 0.6 mm. The overall sensor chip has a square geometry with a side length of 15 mm (Figure S1a). The gas sensor consists of a working electrode on which the active sensing materials 2H MoS_2_@rGO composite, rGO alone, and 1T MoS_2_@rGO composite are deposited (Figure S1b,c). Specifically, the sensing platform adopts a FET‐type device architecture, where the hybrid material functions as the conductive channel between the source and drain electrodes. When exposed to target gases such as NO, NO_2_, and NH_3_, the molecules diffuse into the sensing layer and are adsorbed onto the defect engineered surface. This adsorption induces charge transfer between the gas molecules and the hybrid surface, modulating the local charge density and electronic structure of the channel. This charge redistribution is further illustrated by the charge density difference maps, which visualize electron accumulation and depletion upon gas adsorption. The real‐time resistance variations are monitored by applying a constant DC bias voltage of 1 V across the sensor electrodes during gas exposure, and the resulting resistance is recorded and calculated accordingly.

The FET configuration is intentionally employed due to its intrinsic signal amplification capability, low noise characteristics, and stable room‐temperature operation, which together enable sensitive detection of low‐concentration gases [39]. From a mechanistic perspective, detachment of functional groups during partial reduction generates additional defect edge sites that not only stabilize the metastable 1T phase but also increase the specific surface area of the hybrid structure. These structural features synergistically enhance charge transfer efficiency, improve response intensity, and strengthen selective interactions with nitrogen‐based gas molecules.

Collectively, these synergistic effects demonstrate that defect‐engineered MoS_2_@rGO hybrids are highly promising candidates for reliable, scalable detection of low‐concentration gases.

### 2.2 Fabrication and Characteristics of MoS_2_@rGO Hybrid Composite Material

**FIGURE 2:**
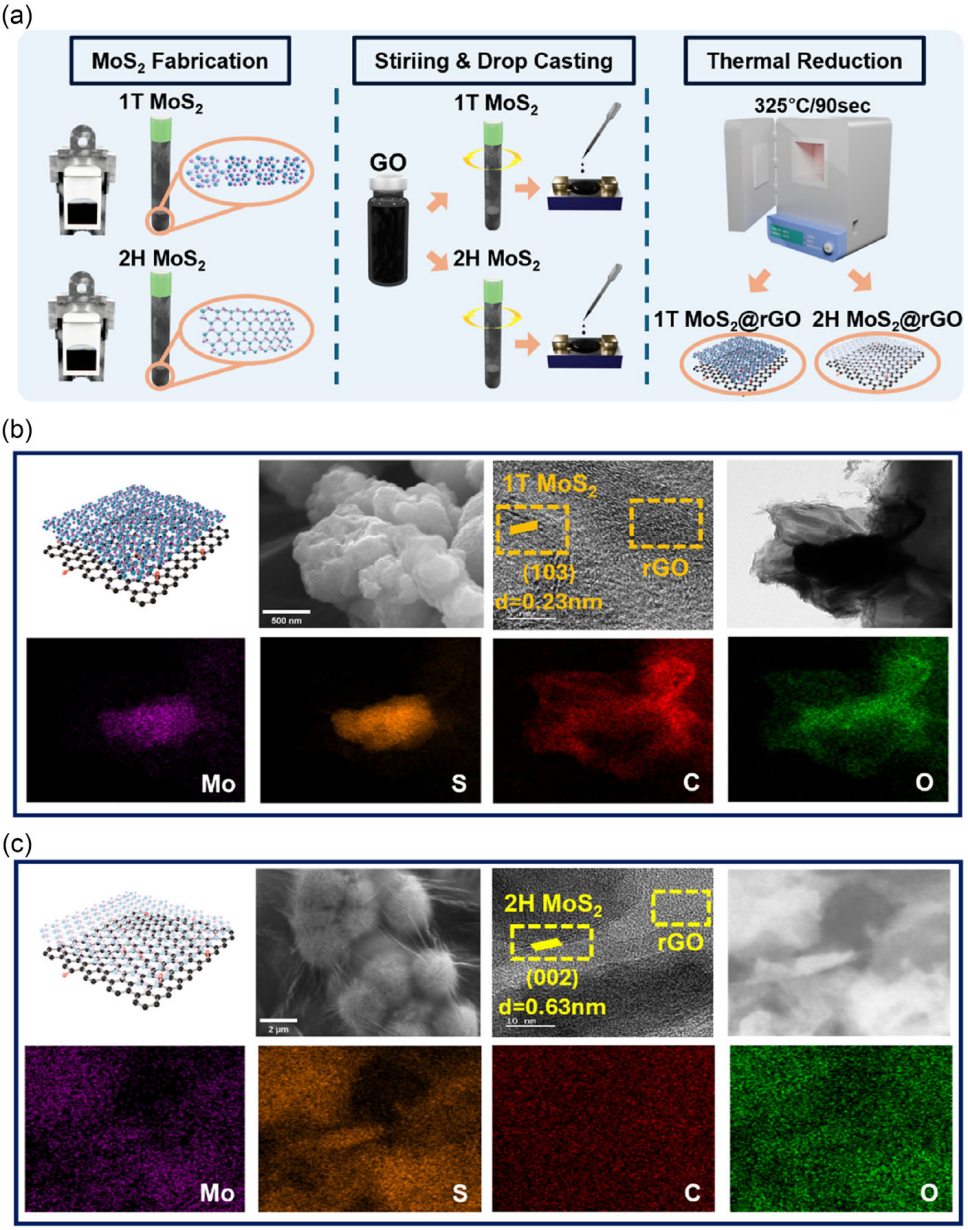
Fabrication: morphological and surface area characterization of rGO‐based hybrid structures. (a) Schematic of the fabrication process for 1T and 2H MoS_2_@rGO. (b) 1T MoS_2_@rGO: crystal model: Field emission scanning electron microscopy (FE‐SEM) image showing aggregated nanosheets, high‐resolution transmission electron microscopy (HRTEM) image with lattice fringes, and energy‐dispersive X‐ray spectroscopy (EDS) elemental mapping. (c) 2H MoS_2_@rGO: crystal model: FE‐SEM image of flower‐like morphology, HRTEM image with lattice fringes, and EDS elemental mapping.

Figure 2a illustrates the synthesis strategy for obtaining the 1T and 2H phases of MoS_2_ on the rGO framework. Successful phase formation was confirmed by XRD, Raman spectroscopy, and X‐ray photoelectron spectroscopy (XPS) analyses. Notably, the use of ammonium molybdate ((NH_4_)_6_Mo_7_O_24_·4H_2_O) at 160°C for 24 h, predominantly promoted the formation of the metastable 1T phase, whereas sodium molybdate (Na_2_MoO_4_·2H_2_O) under harsher conditions of 180°C for 48 h favored the thermodynamically stable 2H phase. These findings demonstrate that both the choice of precursor and hydrothermal conditions play a crucial role in controlling the phase evolution within the hybrid films. For electrode fabrication, MoS_2_ powders were mixed with graphene oxide (GO) in water, sonicated for 20 min, and 2 μL of the suspension was drop‐cast onto the FET channel. The electrodes were then annealed at 325°C for 90 s to enhance structural stability and interfacial contact. This specific thermal reduction condition was intentionally selected to induce partial reduction of GO, rather than complete deoxygenation. This ensured that the essential oxygen‐containing functional groups that facilitate interfacial interactions and enhance the hybrid structure are retained. The resulting degree of reduction is reflected in the structural and surface analyses presented below.

Figure 2b presents the results for 1T MoS_2_@rGO. The SEM image shows a wrinkled and agglomerated sheet‐like structure, whereas the HRTEM image displays the (103) lattice fringes of MoS_2_ (*d* = 0.233 nm) in close contact with the rGO layers. The elemental mapping obtained from EDS confirms the uniform distribution of Mo, S, C, and O, supporting the successful hybridization between MoS_2_ and rGO.

Regarding the 2H MoS_2_@rGO presented in Figure 2c, the FE‐SEM image shows a flower‐like nanostructure, and the HRTEM analysis clearly presents the (002) lattice fringes of 2H MoS_2_ (*d* = 0.63 nm) coexisting with rGO. The EDS mapping results similarly demonstrate that Mo and S are evenly dispersed within the C and O matrix, indicating effective integration of MoS_2_ into the rGO framework.

Overall, Figure 2b,c demonstrate that 1T and 2H MoS_2_ integrated with rGO, exhibit distinct morphological and structural features, confirming the successful formation of the hybrid architecture. These results further highlight the complementary roles SEM, TEM, and EDS analyses play in elucidating the microstructural details of the composites. For pristine 1T MoS_2_, the TEM image and lattice spacing analysis revealed well‐defined lattice fringes, and the EDS mapping confirmed the uniform distribution of Mo and S (Figure S2a). Pristine 2H MoS_2_ exhibits clear (002) lattice fringes along with homogeneous elemental dispersion (Figure S2b). In the case of rGO, the SEM and TEM images along with the EDS mapping clearly demonstrate a wrinkled morphology and carbon‐rich composition (Figure S2c). In conjunction with the observations from Figure S2, these findings collectively support the structural stability and effective integration of each component within the MoS_2_@rGO hybrid system.

To further examine the interfacial characteristics and potential side effects associated with the thermal reduction process, quantitative analyses were conducted using TEM‐derived three‐dimensional reconstructions and EDS‐based elemental mapping (Figure S3a–f). The slope per unit length was calculated to be 0.1191 for 1T MoS_2_@rGO, which is significantly higher than those of 2H MoS_2_@rGO (0.06052) and rGO (0.07720) as shown in Figure S3a–d, indicating more pronounced local height variations and a highly wrinkled yet structurally stable interface rather than thermally induced degradation. Consistently, the ratio of the actual surface area to the projected area was highest for 1T MoS_2_@rGO. Furthermore, spatial entropy analysis revealed that the entropy values of Mo and S distributions in 1T MoS_2_@rGO were approximately two‐ to three‐fold lower than those in 2H MoS_2_@rGO, indicating more homogeneous elemental dispersion. In addition, the Jaccard coefficients were consistently higher for 1T MoS_2_@rGO, demonstrating a greater spatial overlap between Mo and S distributions and confirming robust interfacial integration without adverse effects from the annealing process as shown in Figure S3d–f.

**FIGURE 3:**
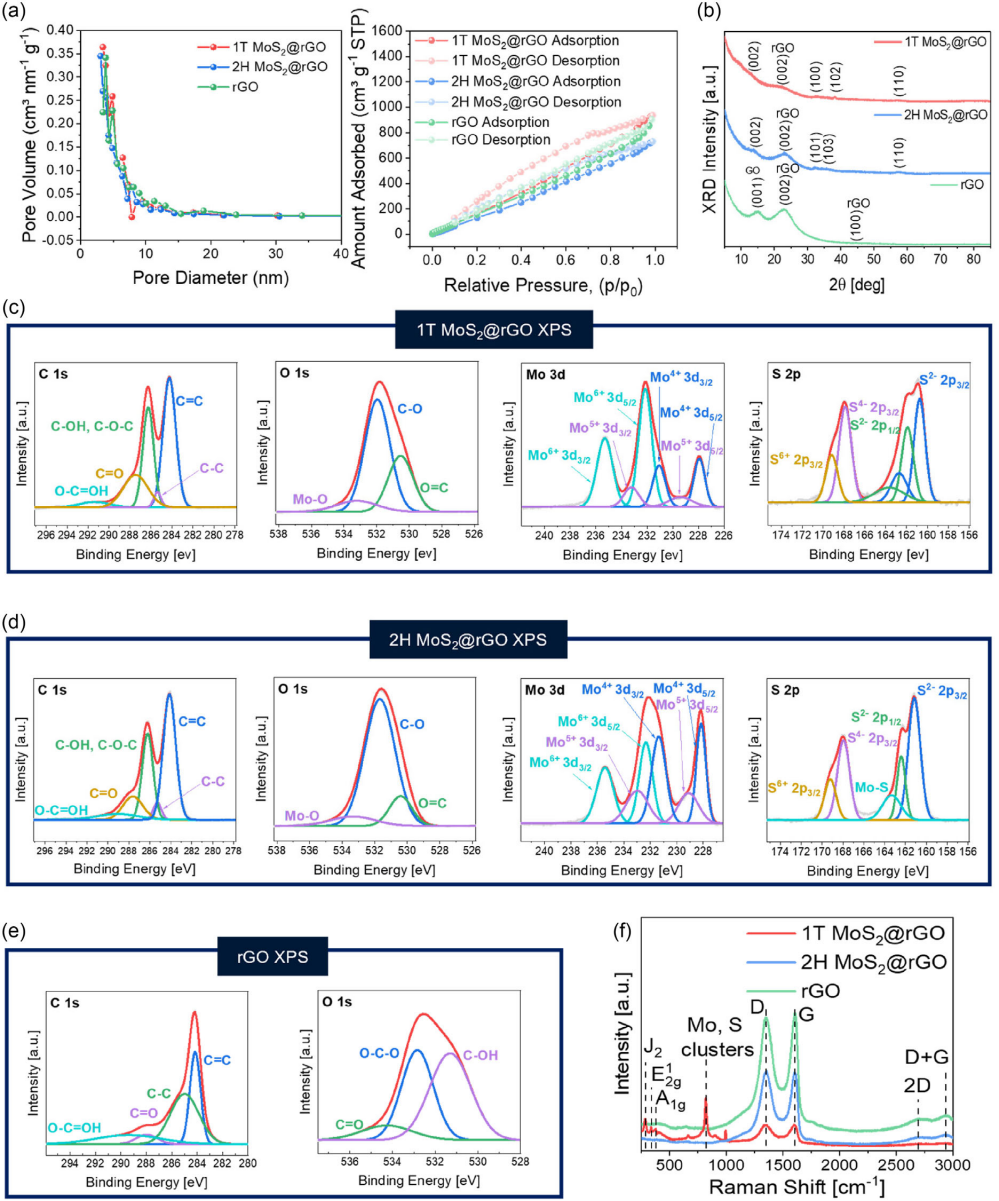
Characterization of MoS_2_@rGO hybrid films**.** (a) Brunauer–Emmett–Teller method (BET)‐based N_2_ adsorption–desorption isotherms and the corresponding Barrett–Joyner–Halenda (BJH) pore size distribution curves of 1T MoS_2_@rGO, 2H MoS_2_@rGO, and pristine rGO, highlighting the mesoporous characteristics and differences in specific surface area and pore volume. (b) XRD patterns of rGO, 1T MoS_2_@rGO, and 2H MoS_2_@rGO. (c,d) The XPS C 1s, O 1s, Mo 3d, and S 2p spectra of (c) 1T MoS_2_@rGO and (d) 2H MoS_2_@rGO. (e) XPS C 1s and O 1s spectra of rGO. (f) Raman spectra of the three samples.

As shown in Figure 3a, the BET analysis confirms that 1T MoS_2_@rGO has the largest pore volume, followed by 2H MoS_2_@rGO and rGO. Both hybrids show isotherms with capillary condensation (H3 type), indicating slit‐like pores from plate‐like aggregation. Pore size distribution shows pores greater than 2 nm, confirming mesoporosity with average diameters of 4.526 nm (1T MoS_2_@rGO), 4.657 nm (2H MoS_2_@rGO), and 4.066 nm (rGO). Such mesoporous structures, together with expanded spacing of 1T MoS_2_@rGO, mitigate restacking, enhance surface area, and provide active sites for gas diffusion beneficial to catalysis and sensing.

The comparative XRD analysis (Figure 3b) shows structural evolution. The rGO exhibits reflections at ∼15° ((001)) and ∼23° ((002)), plus a weak peak at ∼44.5° ((100)), signifying short‐range graphitic order and partial reduction [40, 41, 42]. The 2H MoS_2_@rGO diffractogram shows sharp peaks at 13.6°, 32.3°, 35.6°, and 57.2°, indexed to the crystalline 2H polytype [43, 44, 45]. As the 2H content increases, these peaks become more pronounced (Figure S4). The 1T MoS_2_@rGO shows attenuated intensity and peak broadening at 13.2°, 32.4°, 38.3°, and 57.3°, indicative of smaller crystallite size and disorder [46, 47, 48, 49]. With increasing 1T MoS_2_ content, these peaks are further accentuated (Figure S4). Despite the reduced order, the 1T phase offers superior conductivity, abundant defects, and active edge sites, enhancing electrocatalysis and gas sensing.

Figure 3c–e show the XPS profiles. For 1T MoS_2_@rGO (Figure 3c), the Mo 3d spectrum is broadened with positive shifts (∼228.0, ∼232.2 eV), which is characteristic of 1T MoS_2_ coordination. The Mo^6+^ satellite intensity is enhanced, suggesting oxidation from metastability. The S 2p region shows broadening and shifts, manifesting sulfur vacancies and strained bonds [50, 51]. When the 1T MoS_2_ content increases, these effects intensify, yielding more defects, oxidation, and interfacial bonding (Figure S5a,b). In contrast, 2H MoS_2_@rGO (Figure 3d) shows sharp Mo 3d doublets (228.2, 231.4 eV, Mo^4+^) with a minor satellite (∼235.5 eV, Mo^6+^) and well‐fitted S 2p doublet (161.2, 162.3 eV). Narrow FWHM confirms high purity and crystallinity [52, 53, 54, 55]. As the 2H MoS_2_ content increases, crystallinity and stability are further reinforced (Figure S5c,d ). For rGO (Figure 3e), the C 1s spectrum shows a dominant graphitic peak (∼284.8 eV) with residual oxygen groups (C—O, C=O, O–C=O), confirming partial reduction [56, 57, 58].

Figure 3f shows the Raman spectra. 2H MoS_2_@rGO has sharp peaks at ∼383 ( $E_{2g}^{1}$) and ∼408 cm^−1^ ($A_{1g}$), typical of the semiconducting 2H phase [59]. The 1T MoS_2_@rGO shows a broad J_2_ peak (∼227 cm^−1^) and a weak ∼800 cm^−1^ band from Mo–S clusters, reflecting defects and distortion. Sulfur vacancies are thought to induce local strain and interlayer distortion [60, 61, 62]. With increasing 1T MoS_2_ content, the J modes sharpen and intensify; the Mo–S band strengthens, whereas the rGO D/G bands weaken (Figure S6). All composites show D (∼1350 cm^−1^) and G (∼1580 cm^−1^) bands of rGO. Pristine rGO has the strongest D band, whereas composites show weaker intensity due to MoS_2_ coverage. The 2D (∼2700 cm^−1^) and D + G (∼2930 cm^−1^) bands indicate partial graphitic stacking and π‐conjugation retention [63].

Overall, XPS and Raman analyses corroborate each other, confirming that the 1T MoS_2_ stabilization yields defect‐rich, catalytically active sites. The binding energy shifts in 1T MoS_2_ hybrids, suggesting that the strong interfacial electronic coupling with rGO, enhances charge transfer. The slight increase in Mo 3d binding energy in 1T MoS_2_ indicates reduced core‐hole screening, intrinsic to metallic distortion. These signatures confirm that partial reduction at 325°C/90 s effectively tunes the Fermi level and strengthens interfacial electronic interaction.

### 2.3 Simulation Evaluation of MoS_2_ Based Hybrid Composite Material

**FIGURE 4:**
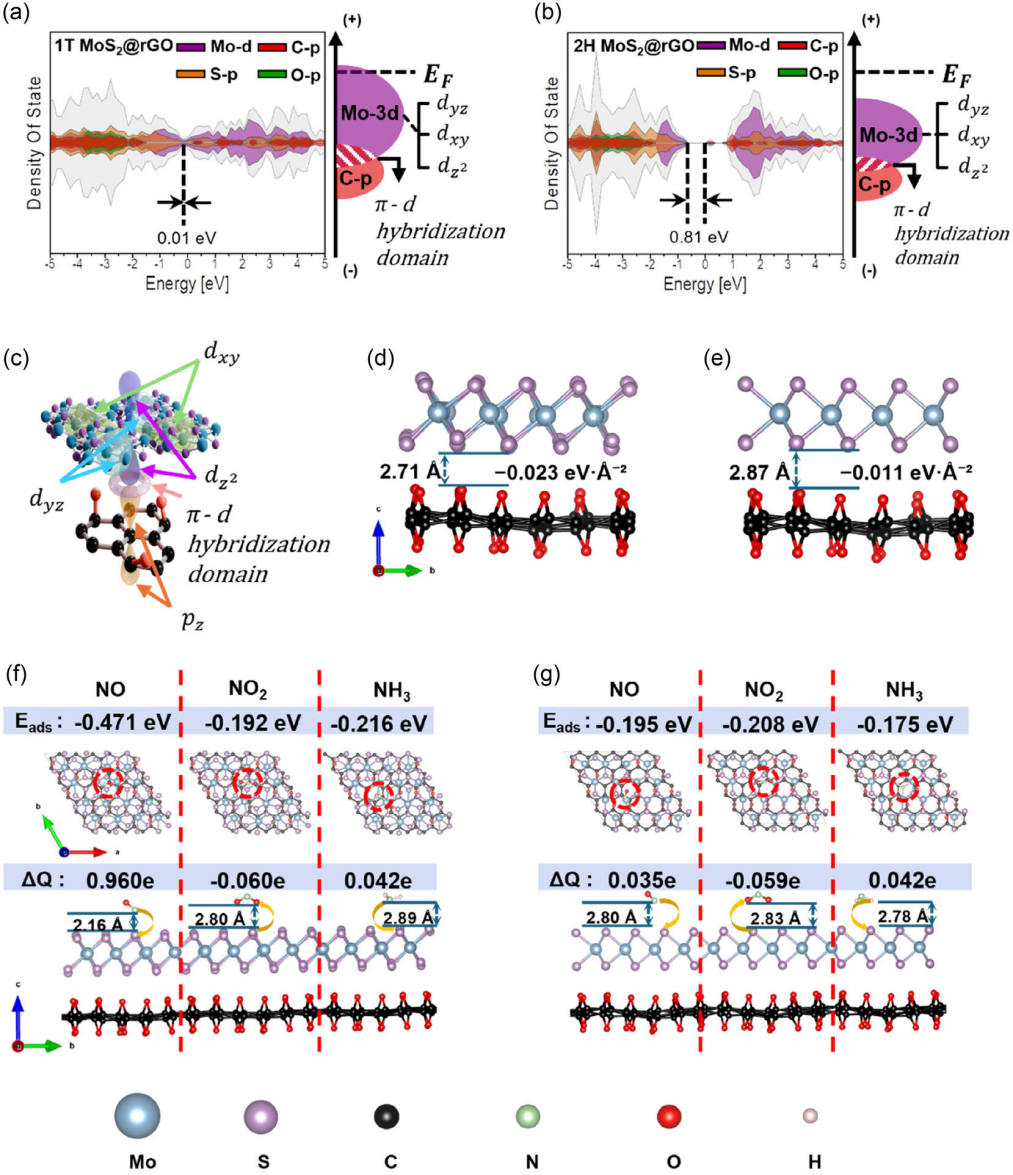
Density functional theory of MoS_2_‐based hybrid active material. Density of states (DOS) of (a) 1T MoS_2_@rGO and (b) 2H MoS_2_@rGO heterostructures and orbital distribution. (c) Schematic of π–d hybridization. Optimized interfacial structure of the (d) 1T MoS_2_@rGO and (e) 2H MoS_2_@rGO heterostructures. Optimized adsorption configurations of NO, NO_2_, and NH_3_ on (f) 1T MoS_2_@rGO and (g) 2H MoS_2_@rGO.

Figure 4a shows the projected DOS (PDOS) of 1T MoS_2_@rGO, where the Mo‐d states remain continuously distributed across the Fermi level and overlap with the C‐2p orbitals of rGO, enabling strong π–d hybridization [64, 65]. In contrast, Figure 4b demonstrates that the 2H MoS_2_@rGO exhibits negligible Mo‐d DOS at the edge of the lowest unoccupied molecular orbital, with the first pronounced Mo‐d peak appearing near +1.0 eV above the Fermi level, which weakens π–d coupling and diminishes hybridization‐driven stabilization, thereby leading to weaker interfacial binding compared to the 1T phase [40]. The π–d hybridization originates from the orbital coupling between Mo‐d states and C‐p orbitals (Figure 4c).

In addition to the interfacial adhesion analysis, the electronic structures obtained from DOS calculations further highlight the effect of rGO on the band gap of MoS_2_. Pristine rGO exhibits a clear band gap of 2.0 eV with no states between 0 and 1 eV (Figure S7a), whereas isolated 1T MoS_2_ and 2H MoS_2_ show gaps of 0.05 and 1.66 eV, respectively (Figure S7b,c). Upon heterostructure formation, the gap narrows significantly: 0.01 eV for 1T MoS_2_@rGO and 0.81 eV for 2H MoS_2_@rGO. Notably, finite DOS contributions from C and O orbitals emerge within the pristine gap region of rGO, indicating the appearance of localized in‐gap states driven by π–d hybridization and charge transfer across the interface. In the 1T heterostructure, the continuous distribution of Mo‐d states at the Fermi level strongly couples with the C‐2p and O‐2p orbitals, producing a quasi‐metallic DOS profile. In contrast, the 2H heterostructure shows weaker but distinct C/O‐derived in‐gap peaks, consistent with moderate band‐gap reduction. These DOS‐based observations demonstrate that interfacial hybridization with rGO not only narrows the band gap of MoS_2_ but also introduces localized electronic states that facilitate enhanced carrier transport.

Figure 4d shows the calculated interlayer binding energy of 1T MoS_2_@rGO (−0.023 eV·Å^−2^), which is more than twice that of the 2H heterostructure (Figure 4e, −0.011 eV·Å^−2^), confirming that both systems are thermodynamically stable, which highlights the stronger adhesion of the 1T phase. Structural relaxation reveals slight octahedral distortion in the 1T phase, whereas the 2H phase retains its trigonal prismatic coordination, providing a structural basis for the enhanced interfacial adhesion observed in 1T MoS_2_.

These theoretical results are consistent with the experimental XPS measurements (Figure 3c–e). For 2H MoS_2_@rGO, the Mo 3d spectrum exhibits sharp peaks at ∼228.2 and ∼231.4 eV, characteristic of Mo^4+^ in trigonal prismatic coordination, along with well‐defined S 2p doublets at ∼161.2 and ∼162.3 eV, confirming preserved crystallinity. In contrast, 1T MoS_2_@rGO displays broader Mo 3d peaks shifted to higher binding energies (∼228.0 and ∼232.2 eV), increased Mo^6+^ contributions, and broadened S 2p features, consistent with distorted octahedral coordination, higher defect density, and stronger interaction with rGO. Both PDOS and XPS analyses consistently demonstrated that the 1T phase possesses more active interfacial sites and stronger adhesion to rGO than the 2H phase.

To evaluate the adsorption energy ($E_{\mathrm{ads}}$), six initial configurations were generated by placing the adsorbate 2 Å above the Mo (metal) and S atoms in both parallel and vertical orientations on the surface. The most stable adsorption geometry is presented in Figure 4.

On the 1T MoS_2_@rGO surface (Figure 4f), the adsorption energies are −0.216 eV (NH_3_), −0.471 eV (NO), and −0.192 eV (NO_2_), whereas on the 2H MoS_2_@rGO surface (Figure 4g) the values are −0.174 eV (NH_3_), −0.195 eV (NO), and −0.208 eV (NO_2_) (Figure S8a). Notably, the 1T MoS_2_@rGO configuration shows a pronounced preference for NO adsorption. Its adsorption energy of −0.471 eV is more than double that of NH_3_ and significantly greater than that of NO_2_. Optimized structural models support this selectivity, with NO stabilizing closer to the surface compared to NH_3_ and NO_2_, in contrast to 2H MoS_2_@rGO with all three molecules adsorbing at nearly identical distances (∼2.8 Å) within a narrow range of adsorption energies. Bader charge analysis confirmed that NO induces the strongest electronic interaction, with the largest charge transfer (ΔQ, +0.096 e), whereas NH_3_ (+0.042 e) and NO_2_ (−0.060 e) exhibited weaker binding and comparable levels of charge redistribution (Figure S8b) [66]. In comparison, charge transfer values for the 2H MoS_2_@rGO heterostructure are smaller: NO (+0.035 e), NH_3_ (+0.014 e), and NO_2_ (−0.059 e) (Figure S8b). This indicates that the semiconducting 2H MoS_2_@rGO is less responsive to adsorption‐induced electronic perturbations, leading to weaker sensing capability. These results highlight the strong selectivity of 1T MoS_2_@rGO toward NO, suggesting its potential as a promising candidate for nitrogen oxide detection.

### 2.4 Gas Response of Partial Reduced Hybrid Active Material Based FET Sensor

**FIGURE 5:**
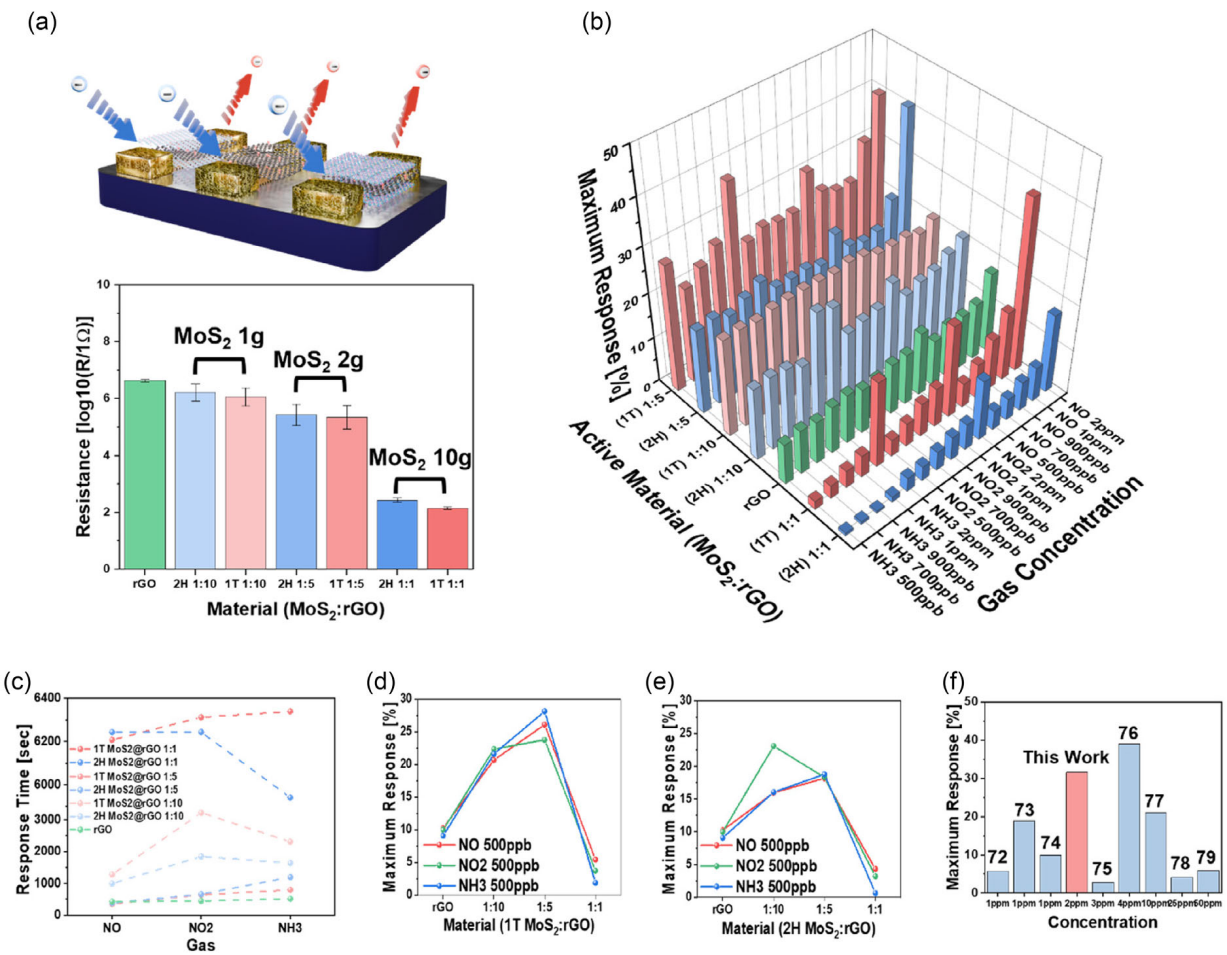
Gas sensing performance of MoS_2_@rGO‐based hybrid materials. (a) Schematic illustration of the electrical operating mechanism of MoS_2_@rGO composites based FET sensor and the baseline resistance values of rGO, 2H MoS_2_@rGO, and 1T MoS_2_@rGO with different mixing ratios (e.g. MoS_2_:rGO). (b) 3D bar plot showing the maximum response of various MoS_2_@rGO sensors for NO, NO_2_, and NH_3_ at different concentrations. (c) Response times of rGO, 1T MoS_2_@rGO, and 2H MoS_2_@rGO sensors to NO, NO_2_, and NH_3_. (d,e) Maximum responses of (d) 1T MoS_2_@rGO and (e) 2H MoS_2_@rGO sensors at different mixing ratios for NO, NO_2_, and NH_3_ (500 ppb). (f) Comparison of the optimized 1T MoS_2_@rGO sensor with previously reported studies, highlighting its superior sensitivity for NO_2_ concentrations ranging from 500 ppb to 2 ppm.

Figure 5a illustrates the schematic configuration of the FET‐type sensor, where a fixed voltage of 1 V is applied to the source electrode and current flows through the electrode coated with the active material toward the drain (Figure S9). The resistance varies depending on the type of active material. With increasing 1T MoS_2_ content, the resistance decreases due to enhanced conductivity, indicating efficient electron transfer facilitated by the 1T phase (Figure S10). Composites of MoS_2_ and rGO were prepared in MoS_2_:rGO ratios of 1:1, 1:5, and 1:10, where the larger proportion of rGO was intentionally chosen to increase the viscosity of the dispersion, thereby enabling uniform deposition of the hybrid material onto the electrode surface. Prior to gas exposure, all sensors were stabilized under flowing dry air to establish a saturated baseline, which was maintained for 10,000 s at a constant flow rate of 500 sccm. All sensing measurements were conducted under identical flow conditions, and for concentration‐dependent measurements, each gas exposure was performed only after the baseline resistance was fully restabilized to ensure reliable and reproducible response evaluation.

A three‐dimensional (3D) bar plot of the maximum response of the sensors for NO, NO_2_, and NH_3_ at different concentrations is presented in Figure 5b.

The response was calculated using the following equation

(1) $$\text{Response }[\%]=\left|\frac{100\times (R-R_{0})}{R_{0}}\right|$$

where *R* denotes the resistance at the point where the change reaches its maximum after gas injection and *R_0_* is the baseline resistance in air. Composites containing 1T MoS_2_ generally exhibit the highest responses, and as the proportion of 1T MoS_2_ increases, the variation in maximum response with gas concentration becomes more pronounced. This trend indicates enhanced sensitivity and selectivity to gas concentration when the 1T phase is enriched. In particular, the sensor shows a markedly higher response to nitrogen‐containing gas molecules, as evidenced by the significantly larger response to NO compared to nonnitrogen‐containing gases shown in Figure S9. This behavior suggests that the defect‐rich and metallic 1T domains promote stronger charge–transfer interactions with nitrogen‐based gas species, thereby enhancing selective sensing performance. Specifically, maximum response of the 1T MoS_2_@rGO (1:1) sensor to NO, H_2_S, and C_2_H_5_OH at 10 ppm which was selected to ensure reliable detection of weakly interacting gases such as C_2_H_5_OH under room‐temperature conditions presented in Figure S11 were obtained approximately 6 months after the primary sensing experiments, using the same sensing layer and device configuration [67]. The consistent response hierarchy observed across different gas species confirms the reproducibility and long‐term stability of the 1T MoS_2_@rGO‐based sensor platform.

Notably, in some 1T‐enriched composites, a higher response is observed at 2 ppm and 500 ppb compared to higher concentrations (700 ppb‐1 ppm), which can be attributed to preferential adsorption at high‐affinity defect and edge sites dominating the low‐concentration regime, resulting in efficient charge transfer and an amplified modulation of the conductive channel [27, 68]. As the gas concentration increases beyond 500 ppb, additional adsorption on lower‐affinity sites together with partial site saturation and charge‐screening effects reduces the effective channel modulation, leading to a diminished sensor response at higher concentrations [69, 70]. In addition, for FET‐type gas sensors, excessive charge transfer at higher analyte concentrations can shift the operating point away from the region of maximum transconductance, further contributing to the observed nonmonotonic response behavior [71].

In Figure 5c, the response rates of the three gases are compared at 500 ppb. Under identical loading of the active material, 1T MoS_2_@rGO demonstrates a faster response to NO compared with 2H MoS_2_@rGO. However, as the MoS_2_ content increases, the response rate becomes slower, which can be attributed not only to stronger binding between the active material and gas molecules but also to an increased overall reaction amount, with both delaying the adsorption–desorption process.

As shown, the maximum responses of 1T MoS_2_@rGO at various mixing ratios are presented in Figure 5d. The maximum response is consistently highest at a ratio of 1:5, with particularly strong enhancement observed for NO gas as the content of 1T MoS_2_ increases. In Figure 5e, 2H MoS_2_@rGO exhibits a similar but less pronounced trend, with responses consistently lower than those of the 1T‐based composites, indicating its relatively inferior performance in gas adsorption and charge transfer.

Finally, Figure 5f compares the optimized 1T MoS_2_@rGO sensor with previously reported NO_2_ sensors [72, 73, 74, 75, 76, 77, 78, 79]. At 2 ppm, the maximum response observed in this study surpasses those of earlier reports. Even in cases where comparable or higher responses were achieved in prior studies, such performance required higher gas concentrations (e.g., 4 ppm) (Table S2). Therefore, the present sensor demonstrates competitive and advantageous performance at a lower concentration of 2 ppm, highlighting its potential for low‐level NO_2_ detection.

Overall, the results presented in Figure 5 clearly demonstrate that incorporating 1T MoS_2_ into rGO substantially enhances the gas sensing performance across multiple analytes. The reduced resistance, higher sensitivity, faster response to NO, and improved selectivity toward different gases all point to the superior role of 1T MoS_2_@rGO compared with 2H MoS_2_@rGO and pristine rGO. Furthermore, the comparison with previously reported studies highlights that the optimized 1T MoS_2_@rGO sensor achieves outstanding performance at lower gas concentrations, thereby underscoring its competitiveness for practical applications in trace‐level gas detection.

In addition, the MoS_2_@rGO based FET sensor can exhibit nonlinear response behavior with increasing gas concentration, which is intrinsic to defect‐rich, charge transfer dominated FET systems. While the present study employs offline analysis, integration of machine learning approaches could enable real‐time monitoring. Notably, the room‐temperature operation at low concentrations, combined with the high charge–transfer efficiency of the 1T phase, supports pattern‐based selectivity and highlights the potential of this platform for scalable, application‐ready gas sensing (Figure S12).

### 2.5 Response Performance Evaluation of Hybrid Material Based Sensor

**FIGURE 6:**
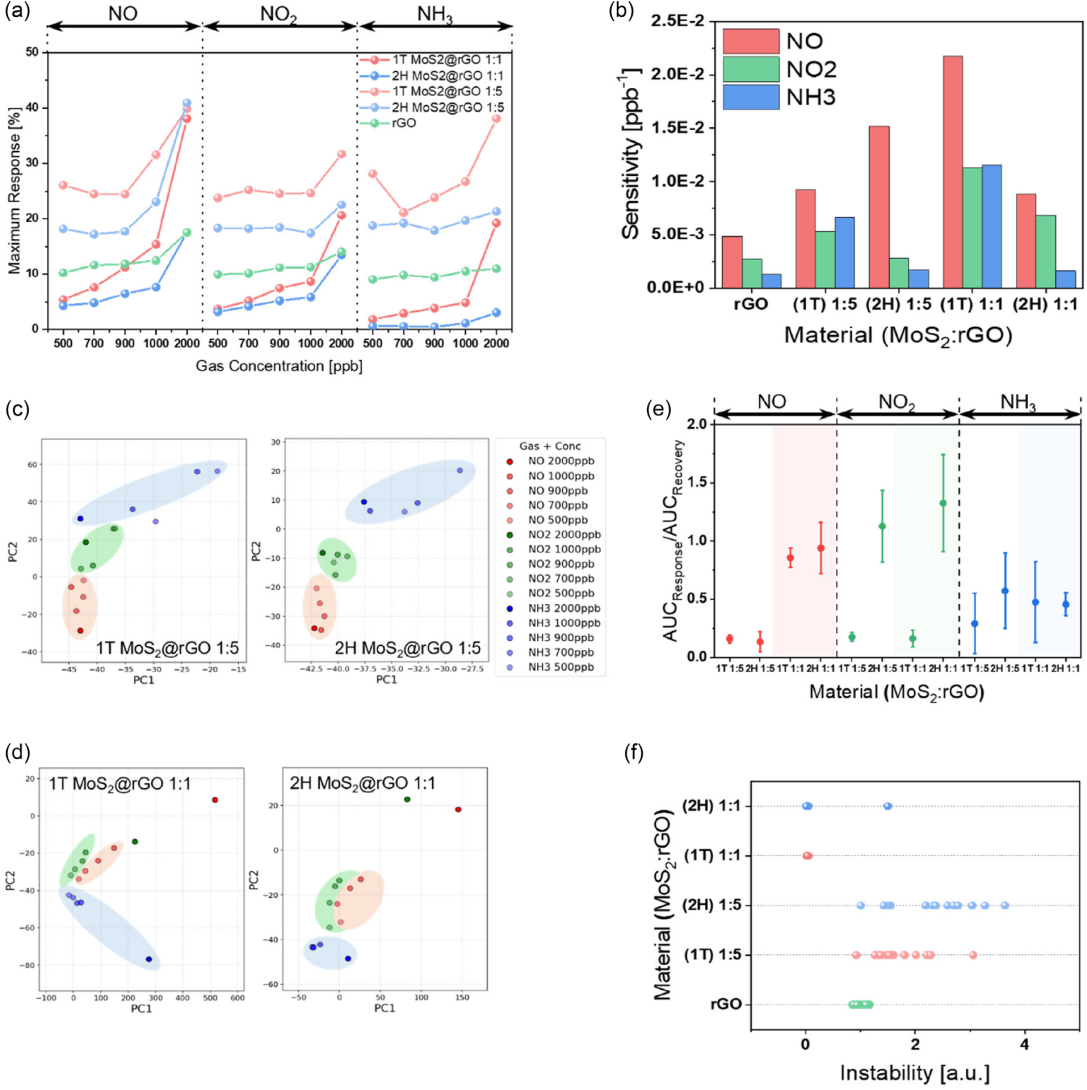
Performance analysis and evaluation of MoS_2_@rGO hybrid sensor**.** (a) Maximum responses of 1T and 2H MoS_2_@rGO and rGO to NO, NO_2_, and NH_3_ at concentrations ranging from 500 ppb to 2 ppm, showing selectivity in materials. (b) Sensitivity values calculated from maximum and minimum responses at 2 ppm and 500 ppb, respectively. (c,d) PCA plot of 1T and 2H MoS_2_@rGO at ratios of (c) 1:5 and (d) 1:1. (e) Ratio of response area under the curve (AUC_response_) to recovery area under the curve (AUC_recovery_) for NO, NO_2_, and NH_3_, comparing 1T MoS_2_@rGO with 2H MoS_2_@rGO at different mixing ratios. (f) Instability analysis of rGO, 1T MoS_2_@rGO, and 2H MoS_2_@rGO, represented by the variation in maximum response.

Figure 6a compares the maximum responses to NO, NO_2_, and NH_3_, revealing that 1T MoS_2_@rGO with a 1:5 ratio exhibits significantly stronger signals than 2H MoS_2_@rGO and pristine rGO. In contrast, at the 1:1 ratio (Figure 6b), the 1T MoS_2_@rGO sample shows a more pronounced dependence on gas concentration, thereby demonstrating superior selectivity toward the target analytes. The sensitivity is calculated as follows

(2) $$\text{Sensitivity}=\frac{{\text{Response}}_{2\;\mathrm{ppm}}-{\text{Response}}_{500\;\mathrm{ppb}}}{2\;\mathrm{ppm}-500\;\mathrm{ppb}}$$

where ${\text{Response}}_{2\;\text{ppm}}$ and ${\text{Response}}_{500\text{ ppb}}$ represent the sensor responses at 2 ppm and 500 ppb, respectively. In analyses, 1T MoS_2_@rGO consistently outperformed rGO across all gases. As the MoS_2_ fraction increased, the 1T based hybrids displayed higher sensitivity than their 2H counterparts, confirming the advantage of the 1T phase in hybrid architectures.

In the principal component analysis (PCA) results of Figure 6c, the clustering of gas types and concentrations is clearly distinguished for the 1:5 composition, with 1T MoS_2_@rGO forming sharper boundaries than 2H MoS_2_@rGO. Similarly, Figure 6d (1:1 composition) shows the same trend, where the 1T based hybrids again exhibit more distinct grouping, confirming their superior discriminative ability for selective vapor detection (Figure S13). The reduced clustering performance observed for the 2H MoS_2_@rGO sensors can be attributed to the intrinsic properties of the 2H phase, namely its semiconducting nature and relatively low defect density. Compared to the metallic and defect‐rich 1T phase, 2H MoS_2_ exhibits weaker charge transfer efficiency and less favorable adsorption energetics toward gas molecules, which limits signal differentiation across analytes and concentrations. To assess the consistency of this behavior, multiple devices were independently tested; specifically, five sensors were fabricated and measured for each composition, and similar clustering trends were observed across all channels.

Figure 6e presents a quantitative comparison of gas response and recovery behaviors. For gas concentrations ranging from 500 ppb to 2 ppm, the areas under the response (AUC_response_) and recovery (AUC_recovery_) curves were calculated, and the mean values were plotted as data points (Figure S14). The results show that in most gas conditions, in response to changes in gas concentration, materials containing 1T MoS_2_ exhibit smaller variations in area ratios than those containing 2H MoS_2_, thereby producing more consistent patterns on the graph. Notably, for NO gas, the area ratio of 1T MoS_2_@rGO at a 1:1 mixing ratio increased by approximately 5.38 fold compared to that at a 1:5 ratio. This finding indicates not only a relative difference in adsorption and recovery kinetics but also a substantial increase in the overall reaction amount at 1:1 compared with the 1:5 condition.

Finally, Figure 6f presents the results of the instability analysis. Instability was evaluated by calculating the standard deviation of the response values within a total of 41 data points centered on the maximum response (${\mathrm{Resp}}_{\max}$) (Figure S15).

The instability is calculated as follows

(3) $$\text{Instability}=\sqrt{\frac{1}{n-1}\sum_{i=1}^{n}{\left({\text{Response}}_{i}-\bar{\text{Response}}\right)}^{2}},n=41$$

where ${\text{Resp}}_{i}$ denotes the response values within the ±20 data points surrounding ${\text{Response}}_{\text{max}}$, and $\bar{\text{Response}}$ is their average. This analysis demonstrates that 1T MoS_2_@rGO samples maintain lower instability values than the 2H‐based analogs, highlighting their improved stability in gas sensing applications. Based on these results, the 1T MoS_2_@rGO hybrid at a 1:1 mixing ratio provides the most favorable performance for NO gas sensing, combining high responsiveness, reliable recovery, and enhanced stability.

## 3 Conclusion

Phase‐changed MoS_2_@rGO hybrids were successfully synthesized and applied to FET type gas sensors capable of operating at room temperature. Structural, morphological, and spectroscopic analyses confirmed that the 1T enriched MoS_2_@rGO hybrid exhibits metallic conductivity, a high density of defects, and abundant exposed edge sites, which collectively form a highly active sensing interface favorable for gas adsorption and efficient charge transfer.

Owing to these phase‐dependent characteristics, the 1T MoS_2_@rGO sensor demonstrated consistently superior sensitivity toward NO, NO_2_, and NH_3_ over a wide concentration range, achieving reliable detection down to sub‐ppm levels at room temperature. Compared with 2H MoS_2_@rGO and pristine rGO based sensors, the 1T hybrid showed sensitivity enhancements by factors of approximately double, underscoring the critical role of phase engineering in tuning the electronic structure and sensing performance.

Furthermore, PCA and signal analyses revealed enhanced selectivity and improved signal stability, confirming reproducible and robust sensing behavior. The clear separation of gas response clusters and reduced signal fluctuations highlight the suitability of the 1T MoS_2_@rGO hybrid for practical sensing environments.

Overall, this work demonstrates that phase‐engineered MoS_2_@rGO is a scalable and effective material platform for low‐concentration nitogen‐based gas vapor detection under ambient conditions, offering strong potential for environmental monitoring and industrial safety applications.

## 4 Experimental Methods

### 4.1 Sensor Electrode Fabrication

An IDE was patterned on a 6‐inch SiO_2_/Si wafer using photolithography. The electrodes were deposited via electron beam evaporation using Ti (10 nm) and Au (50 nm) to form sensing contacts. Each IDE channel comprised 24 electrode fingers with an interelectrode spacing of 5 μm, which helps ensure high surface interaction for sensing applications.

### 4.2 Composite Material Synthesis, Deposition, and Heat Treatment

Hybrid composites of 1T MoS_2_@rGO and 2H MoS_2_@rGO were synthesized via a hydrothermal method using ammonium molybdate ((NH_4_)_6_Mo_7_O_24_·4H_2_O, Sigma–Aldrich) and sodium molybdate (Na_2_MoO_4_·2H_2_O, Sigma–Aldrich) as precursors to preferentially form the metastable 1T phase and the thermodynamically stable 2H phase, respectively.

For preparation of the MoS_2_@rGO composites, physically synthesized 1T MoS_2_ or 2H MoS_2_ powders were mixed with aqueous GO solution (1 wt%, STANDARD GRAPHENE) at controlled ratios: 1 mg MoS_2_ with 1 mL GO (1:10), 2 mg MoS_2_ with 1 mL GO (1:5), and 10 mg MoS_2_ with 1 mL GO (1:1). The mixtures were vortex‐stirred and subsequently sonicated for 20 min to obtain homogeneous suspensions. The resulting suspensions were drop‐cast onto patterned electrodes or substrates.

For pristine rGO sensors, liquid‐phase GO solution (STANDARD GRAPHENE) was drop cast onto patterned electrodes using a micropipette. The deposited GO was stabilized in a desiccator for at least 24 h.

Finally, all fabricated films including MoS_2_@rGO composites and pristine GO layers were prepared by drop‐casting 2 μL of the mixed suspension onto the sensor electrodes, followed by annealing at 325°C for 90 s in an oven (DAIHAN Scientific) to induce partial reduction of GO. This thermal reduction step converted GO into rGO while retaining the oxygen containing the functional groups essential for interfacial interactions and effective gas sensing performance.

### 4.3 Characterization

The structural, morphological, and surface properties of the MoS_2_@rGO composites were comprehensively characterized using a combination of microscopic and spectroscopic techniques. FE‐SEM, (JEOL‐7800F, JEOL Ltd.) was employed to examine surface morphology. For FE‐SEM imaging, the hybrid solutions were drop‐cast onto carbon cloth substrates, dried, and subsequently annealed at 325°C for 90 s. Transmission electron microscopy (TEM, JEM‐F200, JEOL) was used to investigate lattice fringes and microstructural details. TEM samples were prepared by drop‐casting the suspensions onto fabricated chips, followed by drying and annealing at 325°C for 90 s. Additionally, powder samples were dispersed in ethanol, deposited onto TEM grids, dried, and annealed under the same conditions prior to observation.

X‐ray diffraction (XRD, SmartLab, Rigaku) was conducted on powdered samples using over a 2*θ* range of 5°–85° at a scan rate of 5°/min to identify the crystalline phases. Raman spectroscopy (LabRam Aramis, Horiba Jobin Yvon) was performed with a 532 nm laser excitation over a spectral range of 50–3000 cm^−1^. Samples were prepared by drop‐casting onto chips, followed by drying and annealing at 325°C for 90 s. The Raman spectra exhibited the characteristic features of both rGO and MoS_2_.

Surface chemistry and interfacial interactions were analyzed by XPS, (K‐alpha, Thermo Fisher Scientific), focusing on the C 1s, O 1s, Mo 3d, and S 2p regions. Binding energies were calibrated using the C 1s peak at 284.8 eV. Specific surface area and porosity were evaluated using BET analysis with nitrogen adsorption–desorption isotherms at 77 K (Autosorb IQ, Quantachrome Instruments). Pore size distributions were determined using the Barrett–Joyner–Halenda (BJH) method, confirming the mesoporous characteristics of the composites.

### 4.4 Density Functional Theory Section

All density functional theory (DFT) calculations were conducted using the Vienna Ab initio Simulation Package [80] within the projector augmented‐wave formalism [81]. The exchange–correlation interaction was treated by the generalized gradient approximation with the Perdew–Burke–Ernzerhof functional [82]. Long‐range van der Waals interactions were included using the DFT‐D3 correction proposed by Grimme [83]. A kinetic energy cutoff of 520 eV was employed for the plane‐wave basis. The electronic self‐consistency criterion was set to 1.0 × 10^−5^ eV, and ionic relaxations converged when the maximum residual force was less than 0.03 eV/Å. Gaussian smearing with a width of 0.05 eV was applied for Brillouin‐zone integration. The heterostructure models of 1T MoS_2_@rGO and 2H MoS_2_@rGO were constructed by combining a 4 × 4 × 1 MoS_2_ supercell with a 5 × 5 × 1 graphene supercell. The calculated lattice mismatch was 3.89% for 1T MoS_2_ and 3.97% for 2H MoS_2_. A vacuum spacing of 20 Å was introduced along the normal surface to avoid spurious periodic interactions. All atomic positions were fully relaxed while keeping the in‐plane lattice constants fixed to preserve commensurability. Structure optimization and subsequent static calculations including DOS were performed using a 5 × 5 × 1 Monkhorst–Pack k‐point grid.

The interlayer binding energy of MoS_2_@rGO heterostructures (1T MoS_2_@rGO and 2H MoS_2_@rGO) was evaluated as follows:

(4) $$E_{\mathrm{bind}}=\frac{E_{MoS_{2}@rGO}-E_{MoS_{2}}-E_{rGO}}{S}$$

where $E_{\mathrm{bind}}$is the total energy of the heterostructure; $E_{MoS_{2}}$and $E_{rGO}$ are the energies of the isolated layers in the same supercell. The adsorption energy was calculated as follows

(5) $$E_{\mathrm{ads}}=E_{\text{surface}+\text{adsorbate}}-E_{\text{surface}}-E_{\text{adsorbate}}$$

where $E_{\text{surface}+\text{adsorbate}}$, $E_{\text{surface}}$, and $E_{\text{adsorbate}}$ denote the total energies of the adsorbate–surface system, the clean surface, and the isolated adsorbate molecule, respectively. The net electron charge transfer (Δ*Q*) to the adsorbate was defined as follows

(6) $$\Delta Q=Q_{\mathrm{iso}}-Q_{\text{adsorbed}}$$

where $Q_{\text{adsorbed}}$ and $Q_{\mathrm{iso}}$ represent the total Bader charge on the molecule in the adsorbed and isolated states, respectively.

### 4.5 Gas Sensing Measurements

The gas sensing performance was evaluated using a custom‐designed gas sensing jig equipped with IDEs configured in a FET‐type geometry. A constant bias voltage of 1 V was applied between the source and drain electrodes while monitoring the current through the sensing channel in real time. The active materials (rGO, 1T MoS_2_@rGO, and 2H MoS_2_@rGO) with varying mixing ratios (1:1, 1:5, and 1:10) were drop‐cast onto the sensing electrodes, dried, and annealed at 325°C for 90 s prior to testing. During sensing measurements, the target gases (NO, NO_2_, NH_3_) were introduced at a controlled flow rate of 500 sccm using pure air as the carrier gas. The gas concentrations were systematically varied at 500 ppb, 700 ppb, 900 ppb, 1 ppm, and 2 ppm.

For the sensing protocol, the 1T MoS_2_@rGO (1:1) and 2H MoS_2_@rGO (1:1) sensors were first exposed to pure air for 10,000 s to achieve baseline saturation, followed by 7,000 s of target gas injection to induce a response, and then 8,000 s of air purging to allow recovery. In contrast, the 1T MoS_2_@rGO (1:5, 1:10) and 2H MoS_2_@rGO (1:5, 1:10) sensors were first subjected to identical 10,000 s air saturation and subsequently to 3,000 s of gas injection to induce a response and 7,000 s of air exposure for recovery.

The resistance values of each sensing channel were simultaneously monitored using a switch system (Keithley 7001), which sequentially opened each channel of the interface circuit at 1‐s intervals. The transmitted electrical responses from the sensor array were recorded using a source meter (Keithley 2612A), and all resistance signals were logged as sensing outputs via an IEEE‐488 GPIB interface connected to a computer.

To evaluate the long‐term sensing characteristics and discriminate among different gases and concentrations, principal component analysis (PCA) was performed on the response data obtained under various concentrations.

## Supporting Information

Additional supporting information can be found online in the Supporting Information section.

## Author Contributions

**Hye Gyu Cha**: conceptualization (lead), formal analysis (lead), investigation (lead), methodology (lead), resources (lead), validation (lead), visualization (lead), writing – original draft (lead), writing – review and editing (equal). **Taeseo Ko**: formal analysis (equal), software (equal), validation (equal), writing – original draft (equal), writing – review and editing (equal). **Taehyeon Kim**: formal analysis (supporting), investigation (supporting), validation (supporting). **Yun Ji Hwang**: data curation (supporting), formal analysis (supporting), methodology (supporting), validation (supporting), writing – original draft (supporting). **Sushanta Kumar Das**: conceptualization (supporting), formal analysis (supporting), resources (supporting). **Kyoungmin Min**: formal analysis (equal), funding acquisition (equal), supervision (equal), validation (equal), writing – review and editing (equal). **Seong Chan Jun**: conceptualization (lead), funding acquisition (lead), supervision (lead), validation (lead), writing – review and editing (lead).

## Funding

The National Research Foundation of Korea grant funded by the Korea government (RS‐2024‐00339770) (SCJ). Korea Environment Industry & Technology Institute through the Technology Development Project for Biological Hazards Management in Indoor Air Program (or Project) funded by the Korea Ministry of Environment (ARQ202101038001) (SCJ). The National Research Foundation of Korea (NRF) grant funded by the Korea government (MSIT) (RS‐2025‐23523906) (MK).

## Conflicts of Interest

The authors declare no conflicts of interest.

## Supporting information

Supplementary Material

## Data Availability

The data that support the findings of this study are available from the corresponding author upon reasonable request.
