# Supplementary material for: Partially Reduced and Stabilized Phase‐Changed MoS2 Hybrids for Vapor Molecular Absorption at Defects in Edge Sites
Source: Small Sci. 2026 Apr 29;6(5):e202500605. doi: 10.1002/smsc.202500605 (PMC13129413; doi:10.1002/smsc.202500605)
Supplement: Supplementary file 1 — Supplementary Material [file SMSC-6-e202500605-s001.pdf]

Supporting Information

Partially Reduced and Stabilized Phase-Changed MoS_2 Hybrids for Vapor Molecular Absorption at Defects in Edge Sites

Hye Gyu Cha, Taeseo Ko, Tae Hyeon Kim, Yun Ji Hwang, Sushanta Kumar Das, Kyoungmin Min, and Seong Chan Jun**

Hye Gyu Cha, Taeseo Ko, Tae Hyeon Kim, Yun Ji Hwang, Sushanta Kumar Das, Kyoungmin Min, Seong Chan Jun
School of Mechanical Engineering, Yonsei University, 50, Yonsei-ro, Seodaemun-gu, Seoul 03722, Republic of Korea

E-mail: scj@yonsei.ac.kr (SCJ), kmin.min@yonsei.ac.kr (KM)

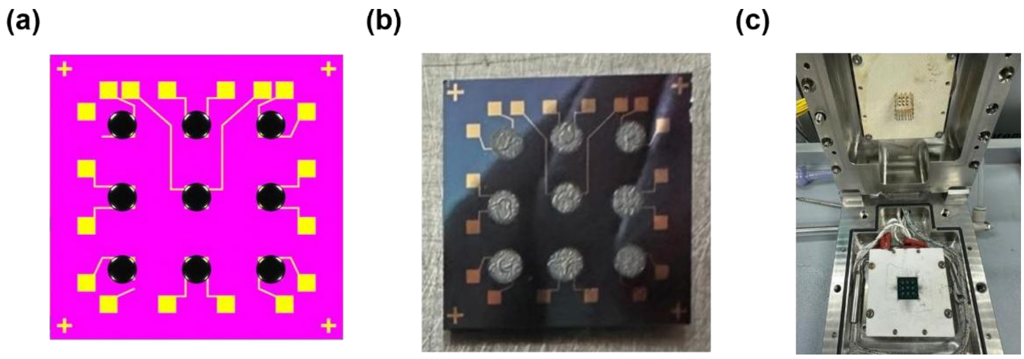

Figure S1. Schematic of fabricated device and measurement setup for the gas sensor. (a) Electrode pattern layout showing interdigitated contacts designed for multiple sensing channels. (b) Photograph of the fabricated sensor chip with deposited sensing material. (c) Experimental setup for gas sensing measurements, with the sensor chip mounted inside the custom gas sensor jig through electrical connections.

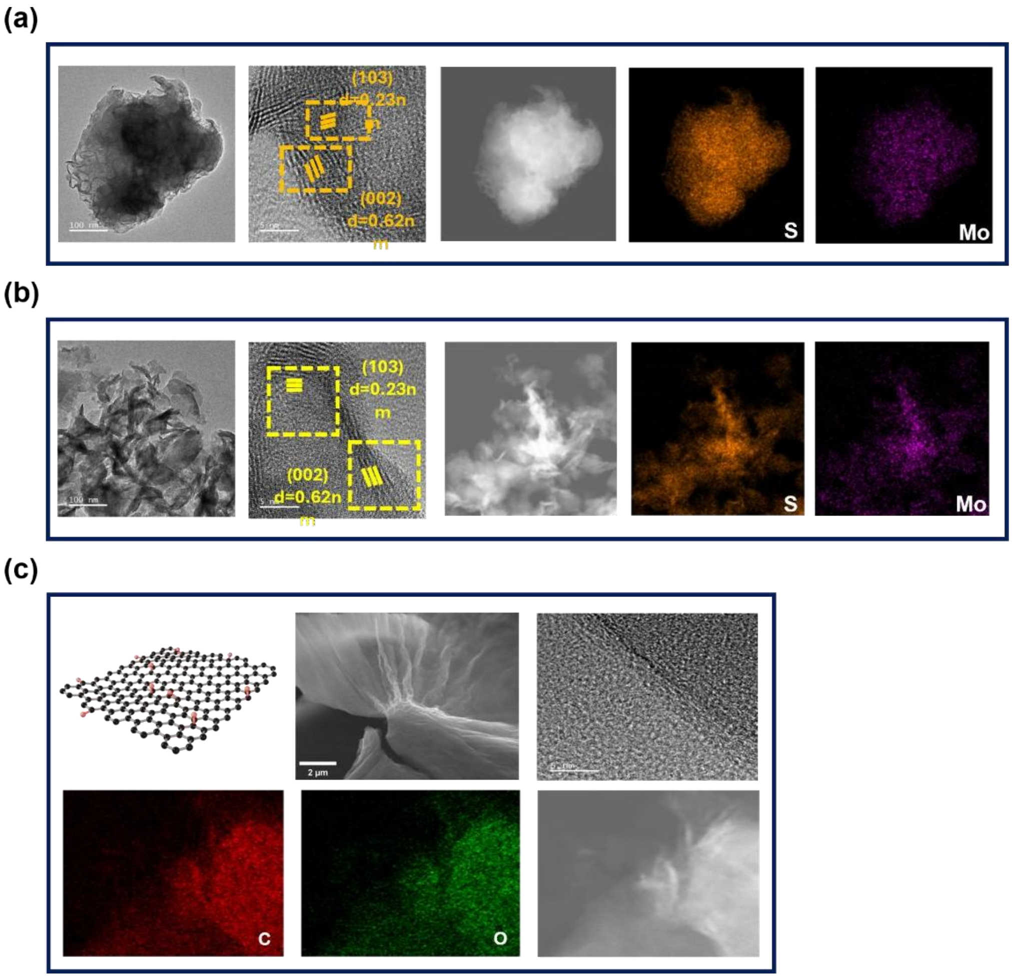

Figure S2. Morphological and elemental analyses of 1T MoS_2, 2H MoS_2, and reduced graphene oxide (rGO). TEM and EDS mapping images of (a) 1T MoS_2, showing uniform distributions of sulfur (S) and molybdenum (Mo) and (b) 2H MoS_2, confirming evenly distributed S and Mo elements within the layered structure. (c) Structural and surface characteristics of rGO: atomic schematic, SEM image, and HRTEM lattice structure. EDS elemental mappings of carbon (C, red) and oxygen (O) further reveal the presence of defect sites and residual oxygen functionalities.

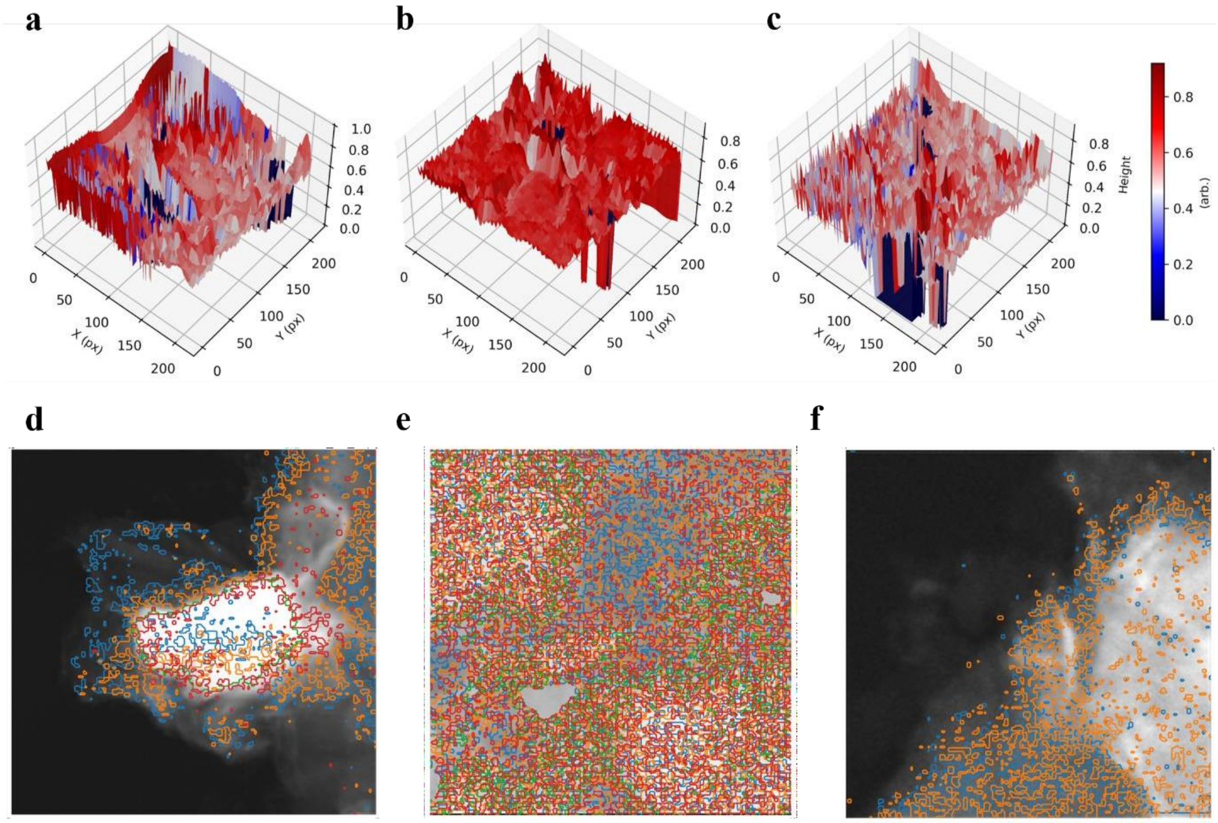

Figure S3. TEM-based structural and elemental distribution analysis; (a–c) Three-dimensional surface representations converted from TEM 2D images based on grayscale contrast, used to evaluate the degree of surface wrinkling for (a) 1T MoS_2@rGO, (b) 2H MoS_2@rGO, and (c) rGO. (d–f) Overlay images of EDS maps extracted from the corresponding TEM images, showing the elemental mixing within the structures for (d) 1T MoS_2@rGO, (e) 2H MoS_2@rGO, and (f) rGO.

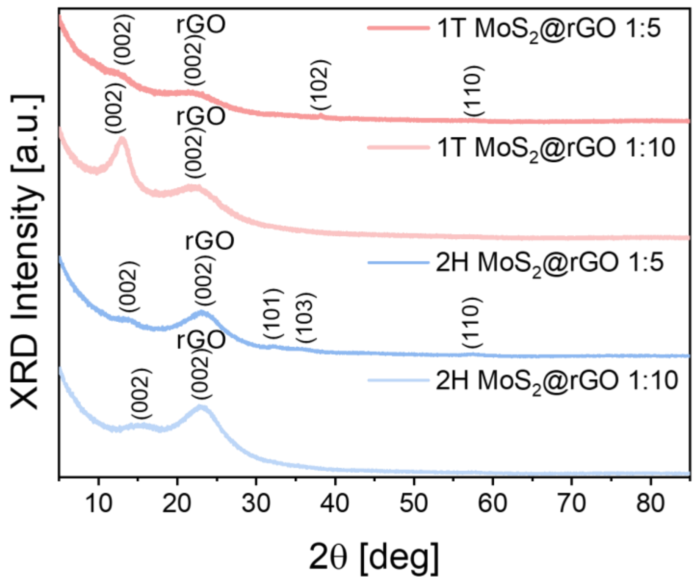

Figure S4. XRD analysis of 1T MoS_2@rGO and 2H MoS_2@rGO with varying MoS_2 content**.** XRD patterns of 1T MoS_2@rGO (1:5), 1T MoS_2@rGO (1:10), 2H MoS_2@rGO (1:5), and 2H MoS_2@rGO (1:10), from top to bottom.

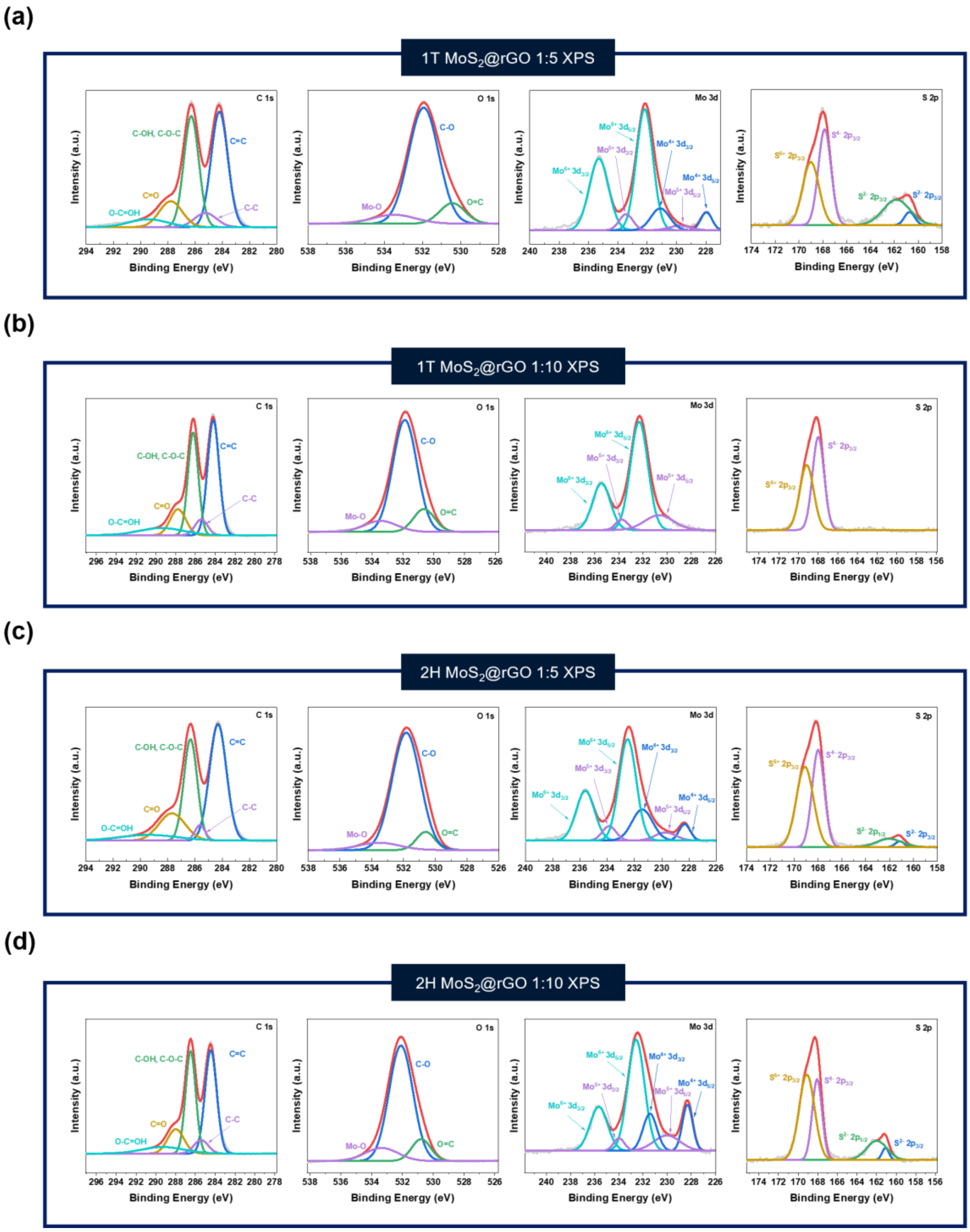

Figure S5. XPS analysis of MoS_2@rGO composites with different MoS_2 contents**.** XPS spectra of (a) 1T MoS_2@rGO 1:5, (b) 1T MoS_2@rGO 1:10, (c) 2H MoS_2@rGO 1:5, and (d) 2H MoS_2@rGO 1:10, showing C 1s, O 1s, Mo 3d, and S 2p peaks. The deconvoluted peaks indicate the chemical states of carbon, oxygen, molybdenum, and sulfur species in the composites.

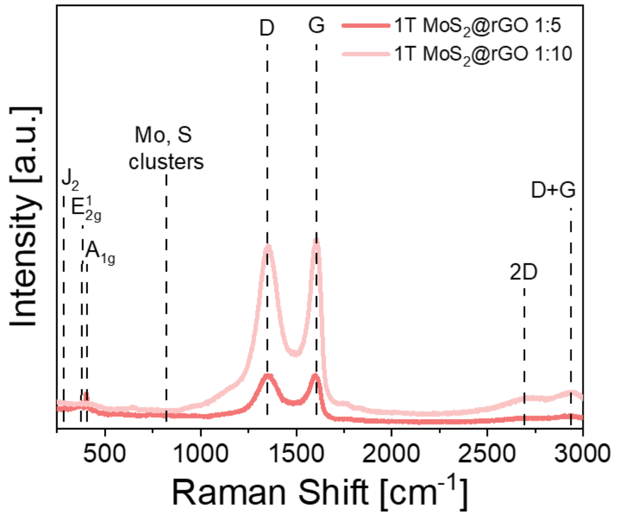

Figure S6. Raman spectra of 1T MoS_2@rGO composites with varying MoS_2 content**.** Raman spectra of 1T MoS_2@rGO (1:5 and 1:10) showing the characteristic peaks corresponding to the in-plane and out-of-plane vibrational modes of MoS_2. Differences in relative intensity and peak positions between the two samples indicate the effects of MoS_2 content and interactions with rGO.

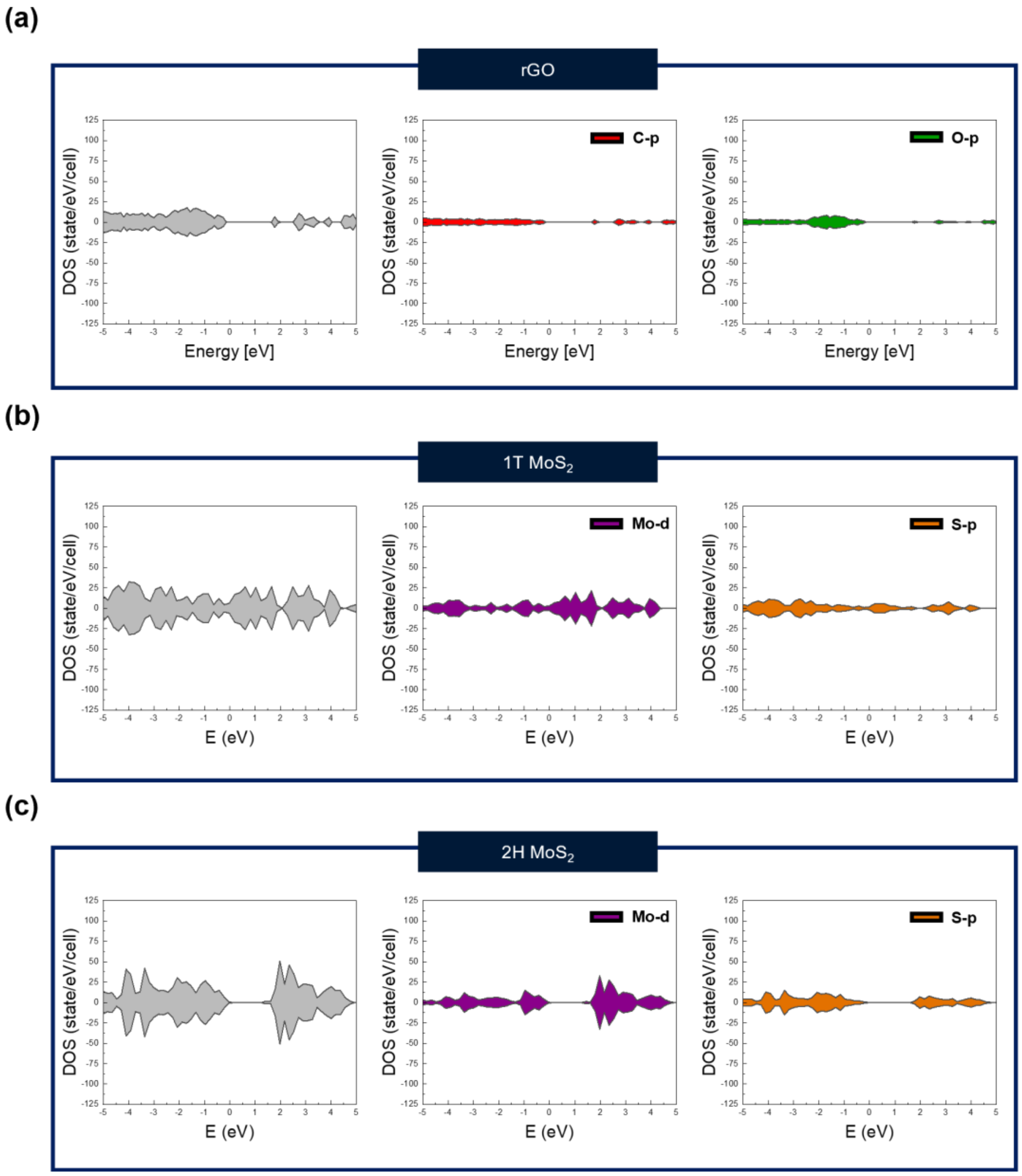

Figure S7. Calculated density of states (DOS)**.** (a) Reduced graphene oxide (rGO), (b) 1T MoS_2, and (c) 2H MoS_2.

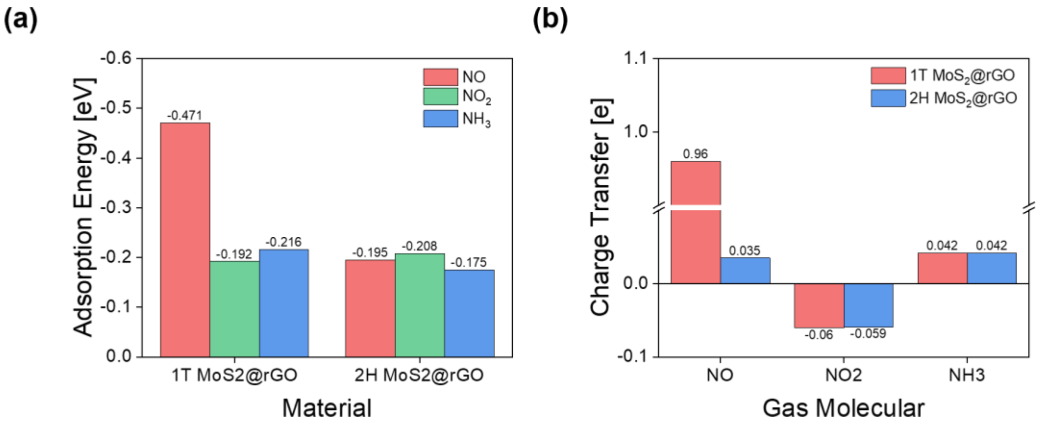

Figure S8. Calculated adsorption energy and charge transfer**.** (a) Adsorption energies of NH_3, NO, and NO_2 on 1T and 2H MoS_2@rGO surfaces. (b) Charge transfer analysis for each gas molecule on 1T and 2H MoS_2@rGO

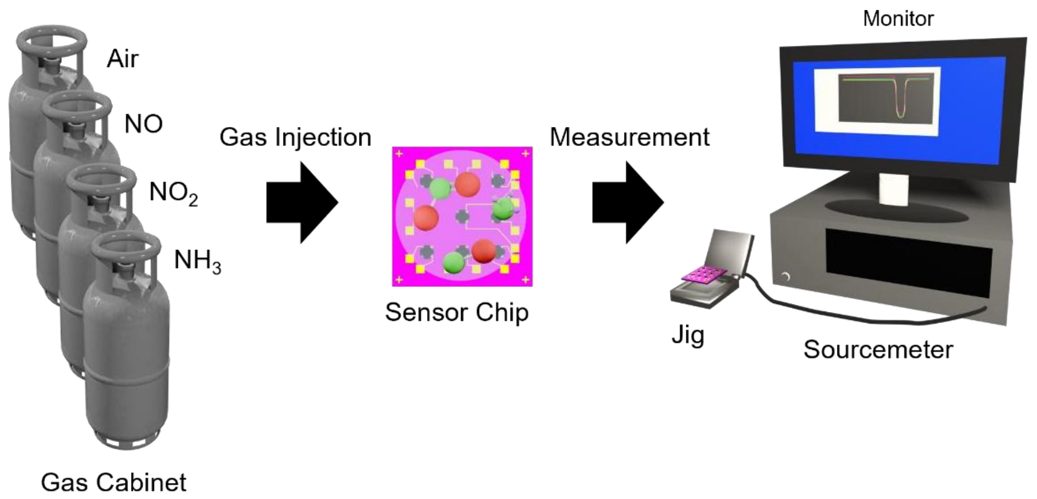

Figure S9. Schematic illustration of the gas sensing measurement system and setup, showing target gas introduction, interaction with the MoS_2@rGO based sensing material, and the corresponding resistance signal monitoring through a computer-controlled system.

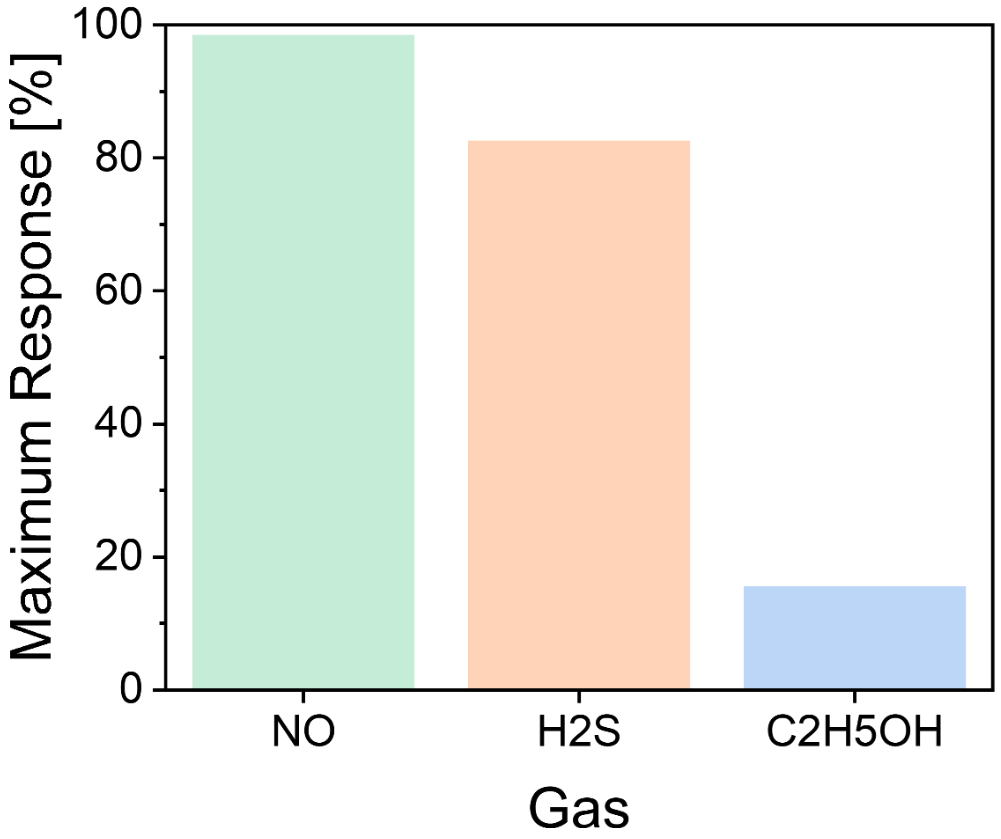

Figure S10. Maximum response of the 1T-MoS_2@rGO (1:1) sensor to NO, H_2S, and C_2H_5OH at 10 ppm

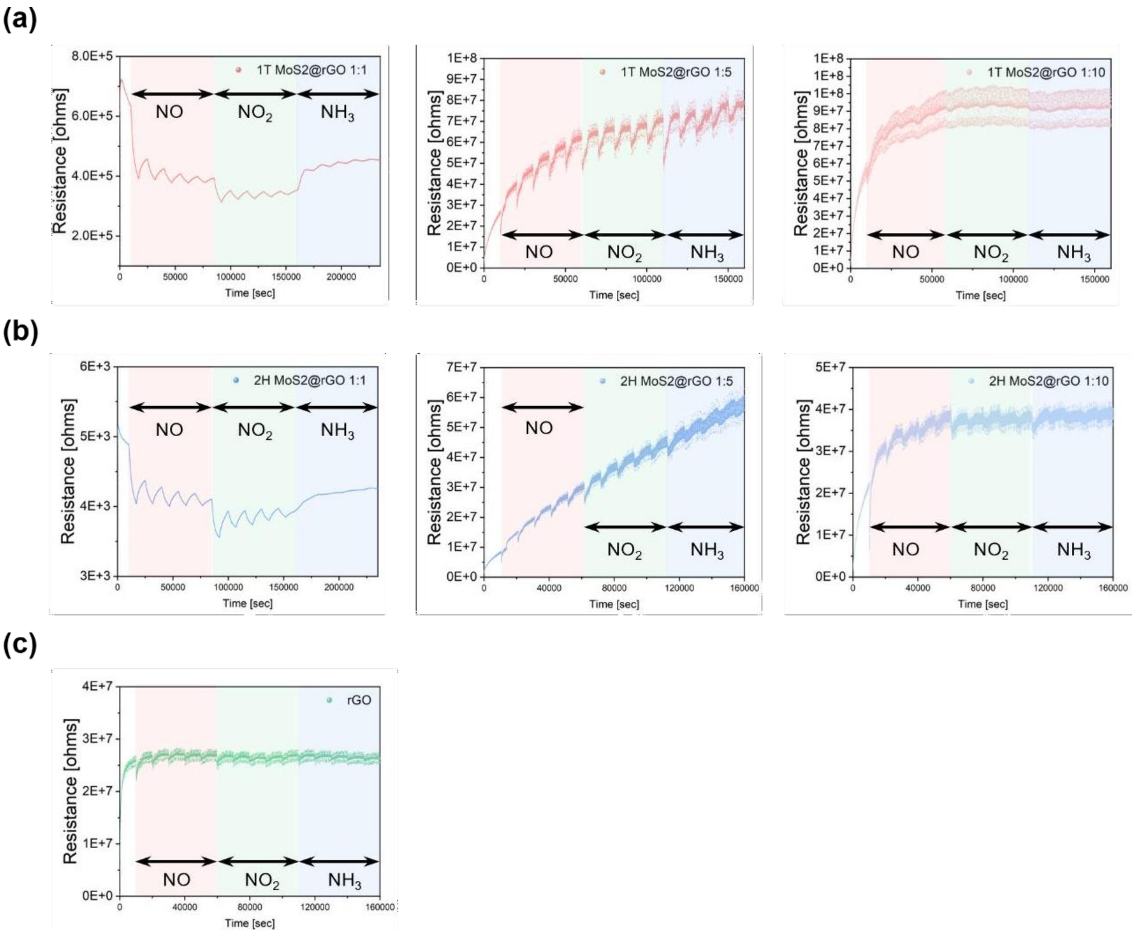

Figure S11. Resistance changes in MoS_2@rGO composites and pristine rGO for different gases (NO, NO_2, and NH_3) at different concentrations. (a) 1T MoS_2@rGO 1:1, 1:5, and 1:10, (b) 2H MoS_2@rGO 1:1, 1:5, and 1:10, and (c) pristine rGO under exposure to NO, NO_2, and NH_3 from 500 ppb to 2 ppm.

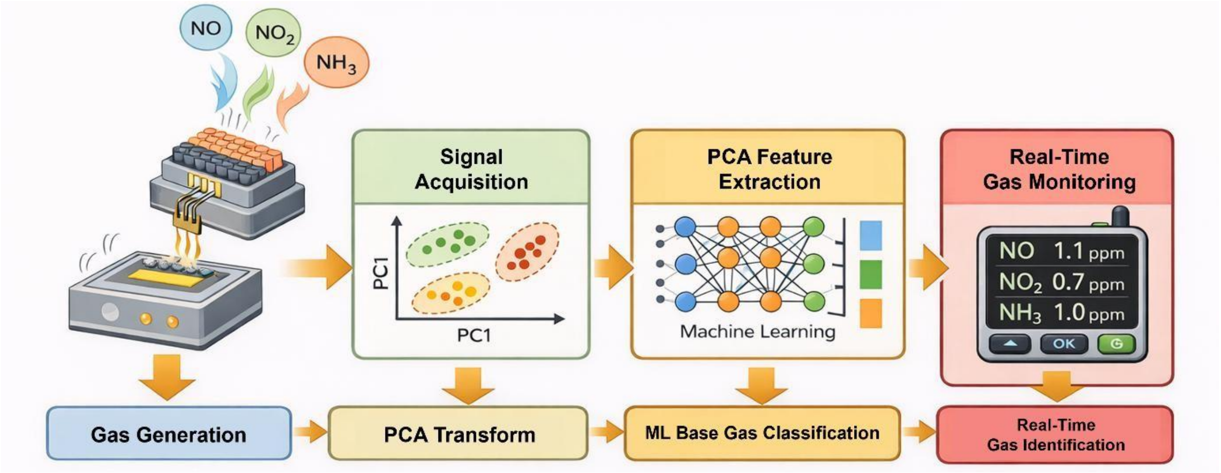

Figure S12. Schematic workflow illustrating the concept of real-time gas monitoring enabled by the proposed sensing platform.

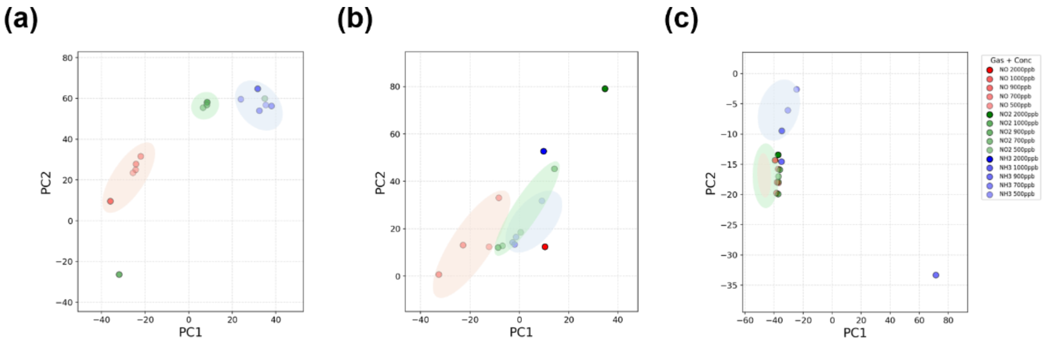

Figure S13. Principal component analysis (PCA) plots of gas sensing responses of MoS_2@rGO composites and pristine rGO for NO, NO_2, and NH_3 at different concentrations (500-2000 ppb). (a) 1T MoS_2@rGO, (b) 2H MoS_2@rGO, and (c) pristine rGO exposed to various concentrations of NO, NO_2, and NH_3.

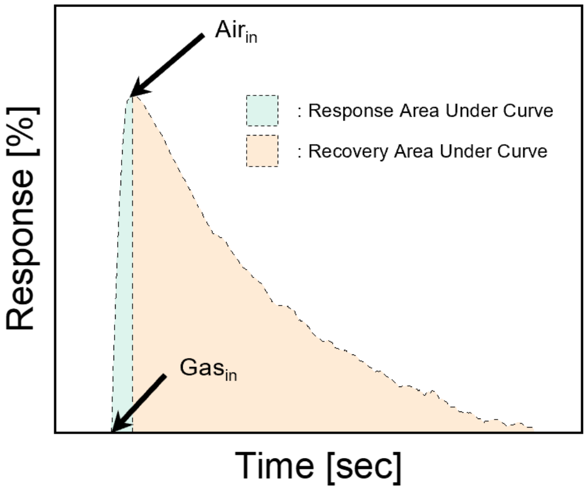

Figure S14. Calculation method of area under the curve (AUC) for gas sensing response. The shaded regions represent the integration areas used to calculate the AUC values for NO and NO_2 exposure. The Gas_{in} point indicates the start of gas introduction, whereas the Air_{in} point denotes the end of gas injection and beginning of air purging.

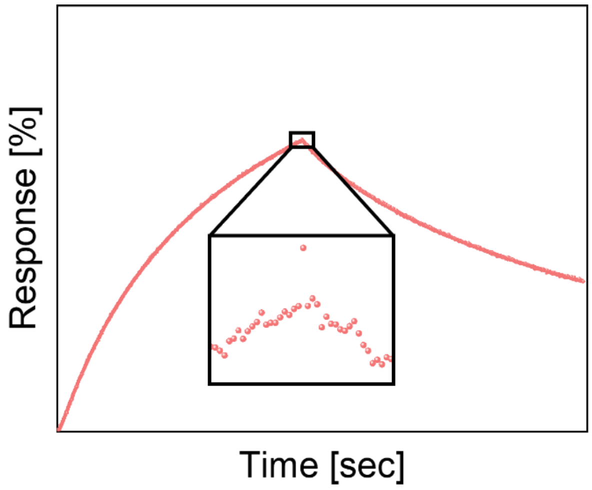

Figure S15. Gas sensing response instability calculation method. Instability was evaluated by calculating the standard deviation of the response using 41 data points, corresponding to 20 indices before and 20 indices after the maximum response point.

Table S1. Representative real-world deployment scenarios and required low-ppm detection ranges for nitrogen-based gases

Scenario	Environment	Target Gas	Mandatory Detection Concentration	Ref.
Semiconductor/Display	Cleanroom	NO_2	~0.1 - 1 ppm	[1]
Chemical Plant	Process Facility	NH_3	≤ 0.5 ppm	[2]
Agriculture/Livestock	Storage/barn	NO_x / NH_3	0.5 - 2 ppm	[3]
Urban Infrastructure	Tunnel/Parking	NO_2	~0.1 - 1 ppm	[1]

Table S2. Gas sensing performance comparison between this work and previous studies.

**Num.**	**Active Material**	**Operating Temp. [°C]**	**Gas Type**	**Concentration [ppm]**	**Response [%]**	**LOD [ppm]**	**Ref.**
1	1T MoS_2@rGO	RT	NO_2	2	20.62	0.5	This work
2	EA-SnO2-rGO	RT	NO_2	1	5.80	1	[4]
3	2H MoS2/MoO3	RT	NO_2	1	18.90	0.05	[5]
4	rGO/Bi2S3	RT	NO_2	1	9.80	0.025	[6]
5	MoS2 nanoflake	RT	NO_2	3	2.82	3	[7]
6	2H WSe2/rGO	RT	NO_2	4	39	0.006	[8]
7	2H MoS2 Bi-layer	RT	NO_2	10	21	10	[9]
8	Mo2C-rGO	RT	NO_2	25	4.10	25	[10]
9	MoS2/rGO@NF	RT	NO_2	50	5.9	50	[11]

REFERNCES

1. Gao, W.; Wang, X.; He, Y.; Yu, H.; Zheng, Y.; Yin, R.; Jiang, X., *Sensors and Actuators B: Chemical* **2024,** *405*, 135350.
2. Park, J. Y.; Lee, J. W.; Cho, H.-B.; Myung, N. V.; Choa, Y.-H., *Sensors and Actuators B: Chemical* **2025**, 138831.
3. Choudhari, U.; Jagtap, S., *Nano-structures & Nano-objects* **2023,** *35*, 100995.
4. Zhang, Y.; Xing, Y.; Yang, Z.; Zhao, L.; Xin, C.; Wei, Z.; Fei, T.; Liu, S.; Zhang, T., *Materials Today Communications* **2024,** *38*, 108090.
5. Yan, J.; Wang, Y.; Yang, C.; Deng, H.; Hu, N., *Journal of Alloys and Compounds* **2024,** *976*, 173208.
6. Yang, Y.; Zhu, M.; Zhang, H.; Wang, B.; Chen, C.; Li, J.; Wang, Y.; Hao, J., *Chemical Engineering Journal* **2024,** *490*, 151872.
7. Gorthala, G.; Ghosh, R., *IEEE Sensors Journal* **2022,** *22* (15), 14727-14735.
8. Shakya, P.; Khan, M. S.; Kumar, S.; Jhankal, D.; Verma, N.; Sachdev, K., *IEEE Sensors Journal* **2025**.
9. Qi, M.; Huang, Z.; Zheng, H.; Zhao, L.; Jiang, R.; Wang, J.; Hu, J.; Chen, G.; Jia, S.; Wang, J., *ACS Applied Nano Materials* **2023,** *6* (11), 9290-9297.
10. Gorthala, G.; Ghosh, R., *IEEE Sensors Letters* **2023,** *7* (12), 1-4.
11. Zhang, H.; Ou, K.; Guan, R.; Cao, Y.; Sun, Y.; Li, X., *Current Nanoscience* **2023,** *19* (3), 401-409.
